# NCI-H295R, a Human Adrenal Cortex-Derived Cell Line, Expresses Purinergic Receptors Linked to Ca^2+^-Mobilization/Influx and Cortisol Secretion

**DOI:** 10.1371/journal.pone.0071022

**Published:** 2013-08-08

**Authors:** Haruhisa Nishi, Hirokazu Arai, Toshihiko Momiyama

**Affiliations:** Pharmacology, The Jikei University School of Medicine, Tokyo, Japan; Indiana University School of Medicine, United States of America

## Abstract

Purinergic receptor expression and involvement in steroidogenesis were examined in NCI-H295R (H295R), a human adrenal cortex cell line which expresses all the key enzymes necessary for steroidogenesis. mRNA/protein for multiple P1 (A_2A_ and A_2B_), P2X (P2X_5_ and P2X_7_), and P2Y (P2Y_1_, P2Y_2_, P2Y_6_, P2Y_12_, P2Y_13_, and P2Y_14_) purinergic receptors were detected in H295R. 2MeS-ATP (10–1000 µM), a P2Y_1_ agonist, induced glucocorticoid (GC) secretion in a dose-dependent manner, while other extracellular purine/pyrimidine agonists (1–1000 µM) had no distinct effect on GC secretion. Extracellular purines, even non-steroidogenic ones, induced Ca^2+^-mobilization in the cells, independently of the extracellular Ca^2+^ concentration. Increases in intracellular Ca^2+^ concentration induced by extracellular purine agonists were transient, except when induced by ATP or 2MeS-ATP. Angiotensin II (AngII: 100 nM) and dibutyryl-cyclic AMP (db-cAMP: 500 µM) induced both GC secretion and Ca^2+^-mobilization in the presence of extracellular Ca^2+^ (1.2 mM). GC secretion by AngII was reduced by nifedipine (10–100 µM); whereas the Ca^2+^ channel blocker did not inhibit GC secretion by 2MeS-ATP. Thapsigargin followed by extracellular Ca^2+^ exposure induced Ca^2+^-influx in H295R, and the cells expressed mRNA/protein of the component molecules for store-operated calcium entry (SOCE): transient receptor C (TRPC) channels, calcium release-activated calcium channel protein 1 (Orai-1), and the stromal interaction molecule 1 (STIM1). In P2Y_1_-knockdown, 2MeS-ATP-induced GC secretion was significantly inhibited. These results suggest that H295R expresses a functional P2Y_1_ purinergic receptor for intracellular Ca^2+^-mobilization, and that P2Y_1_ is linked to SOCE-activation, leading to Ca^2+^-influx which might be necessary for glucocorticoid secretion.

## Introduction

Extracellular nucleotides and nucleosides play important roles in various vertebrate cells and tissues via purinergic (including pyrimidinergic) receptors expressed on the cell surfaces. The functional receptors for the extracellular purines (and pyrimidines) in vertebrates are classified into three P1 G protein-coupled receptors (A_1_, A_2A_, A_2B_, A_3_) for adenosine, eight G protein-coupled P2Y receptors for nucleotides (P2Y_1, 2, 4, 6, 11, 12, 13,14_), and seven ion channel-gated P2X nucleotide receptors for extracellular ATP (P2X_1, 2, 3, 4, 5, 6, 7_) [1], [2], [3].

Purinergic receptors in the adrenal gland (P1 receptors) linked to mineral corticoid secretion are well characterized in rats [4], [5], [6]. In other types of animal cells, especially in bovine adrenal zona fasciculata cells (BAFC), there are many reports of functional P2Y receptors for glucocorticoid secretion. Extracellular ATP [7] and UTP (uridine-5′-triphosphate) [8] induce glucocorticoid secretion in BAFC, and the subtype of the purinergic receptor in this cell type was identified as P2Y_2_ [9]. This dominance of P2 over P1 for steroidogenesis is different from that seen in rats. Furthermore, BAFC are unique in that low concentrations of extracellular ATP, at levels that have no steroidogenic effect alone, enhance ACTH-induced glucocorticoid secretion synergistically [10]. Thus the P2 receptors expressed on adrenocortical cells may have something to do with defense systems under stressful conditions.

As in BAFC, whose steroidogenic enzyme is closer to the human type than that of the rodent, a human-derived adrenocortical cell line, NCI-H295R cell (H295R) [11], expresses all the key enzymes for adrenal steroidogenesis, and produces three steroids (mineral corticoid, glucocorticoid, and DHEA) as functional markers. There are many reports of steroidogenesis in H295R [12], [13], [14], [15], [16] or in human tissues [17], [18], [19]. However, little is known about purinergic receptors inhuman or human-derived adrenocortical cells. Therefore, the present study was focused on elucidating purinergic receptor expression, and their linkage to second messenger systems in H295R.

In the present study, we have shown that H295R express multiple purinergic receptors, and they are functional for intracellular Ca^2+^-mobilization. Furthermore, P2Y_1_ is linked to glucocorticoid secretion. We also have shown that Ca^2+^ influx is required for glucocorticoid secretion in H295R.

## Results

### Expression of P1 and P2 Receptors

**Figure 1:**
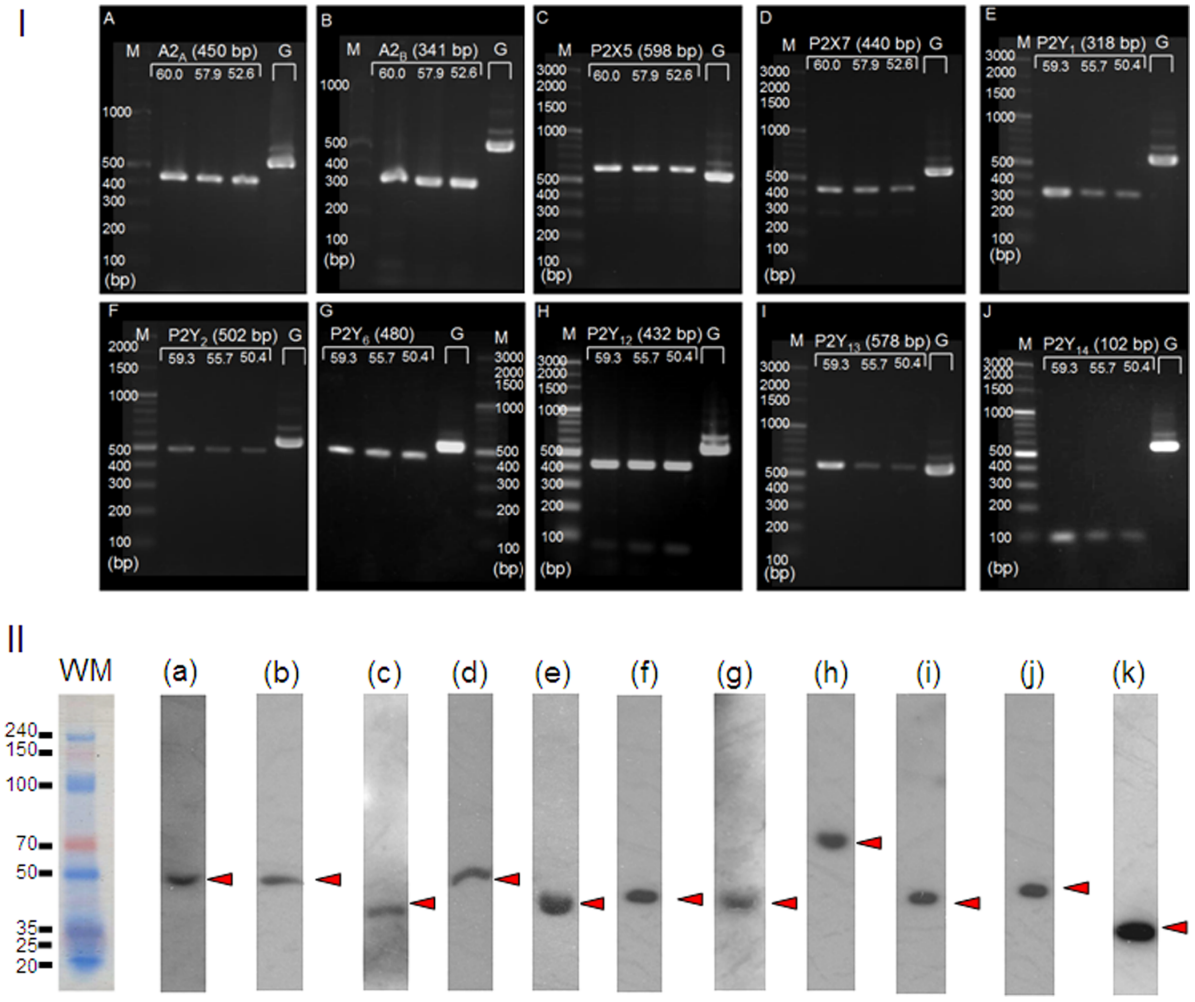
Detection of mRNA/protein for purinergic receptors in H295R. I) mRNA, mRNA for A_2A_ (**A**), A_2B_ (**B**), P2X_5_ (**C**), P2X_7_ (**D**), P2Y_1_ (**E**), P2Y_2_ (**F**), P2Y_6_ (**G**), P2Y_12_ (**H**), P2Y_13_(**I**), and P2Y_14_(**J**) were identified. Each image shows the PCR product bands at three different annealing temperatures, depending upon the melting temperatures (Tm) of the primers. The PCR product amplified for GAPDH (annealing temperature of 58°C) was also loaded on the same gel for each target. The lanes in each gel image show: molecular markers (M), three PCR products for the mRNA target primers (products in high, middle, and low annealing temperatures are indicated), and one using a GAPDH primer (G). Each value in parentheses indicates the molecular weight (bp) for the expected PCR product. The numbers on the three lanes of the targets represent the annealing temperatures used in the individual procedures. II) protein Anti-human antibodies for purinergic receptors were used to confirm the protein expression of the purinergic receptors for which mRNA was detected by PCR in Figure 1–I. The followings are the target proteins and their predicted molecular weights; a: A_2A_ (45 kDa), **b:** A_2B_ (45 kDa), **c:** P2X_5_ (47 kDa), **d:** P2X_7_ (69 kDa), **e:** P2Y_1_ (42 kDa), **f:** P2Y_2_ (42 kDa), **g:** P2Y_6_ (36 kDa), **h:** P2Y_12_ (39 kDa), **i:** P2Y_13_, (41 kDa), **j:** P2Y_14_, (39 kDa), **k:** GAPDH (37 kDa). Molecular sizes (kDa), estimated by pre-stained weight marker, are shown on the left and the right sides of the Figure 1–II. Each arrow head indicates the signal band that is clear and nearest to the predicted size of the target protein.

PCR analysis was carried out to detect mRNA for purinergic receptors in H295R; mRNA for P1 receptors (A_2A_ and A_2B_), P2Y receptors (P2Y_1_, P2Y_2_, P2Y_6_, P2Y_12_, P2Y_13_, and P2Y_14_), and P2X receptors (P2X_5_ and P2X_7_) were detected (Fig. 1–I). Thus, compared to BAFC [20], H295R express various subtypes of purinergic receptors. However P2Y_14_ had not been cloned when the BAFC data were reported [20], and, except for P2Y_1_, the primers used for RT-PCR assays in BAFC were constructed for human message. It is unknown whether the primers for human message matched the mRNA in bovine or BAFC; the latter have no mRNA for these P2Y receptors.

Western blotting was carried out to confirm protein expression of the purinergic receptors for which mRNA was detected in Figure 1–I. All of the purinergic receptors shown in Figure 1–I expressed protein (Fig. 1–II).

### Glucocorticoid Secretion Assay

**Figure 2:**
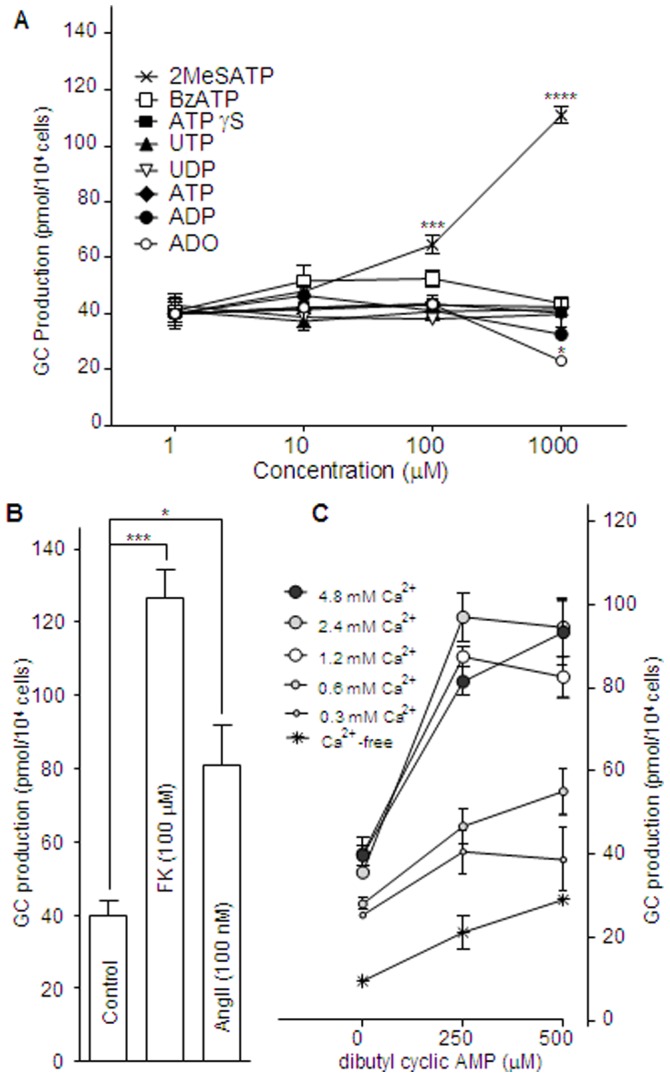
Effects of several purinergic agonists or stimulants on glucocorticoid secretion in H295R. **A:** Effect of ATP on P2X_5_, P2X_7_, and P2Y_2_; ADP on P2Y_1_ and P2Y_13_; UTP on P2Y_2_; UDP on P2Y_6_; 2MeS-ATP on P2Y_1_ and P2X_5_; BzATP on P2X7; ATPγS on P2Y_2_; and, adenosine (ADO) on A_2_A and A_2B_ receptors. **B:** Effects of forskolin and angiotensin II (AngII) on glucocorticoid secretion in H295R. **C:** Effects of extracellular Ca^2+^ concentrations of 0, 0.3, 0.6, 1.2 (standard), 2.4, and 4.8 mM on db-cAMP-induced glucocorticoid secretion in H295R. The cells were incubated at 37°C for 48h. Each point represents the Mean±SE (N = 4). The ‘*’, ‘**’, ‘***’, and ‘****’ indicate statistical significance at *p*<0.05, *p*<0.01, *p*<0.001, and *p*<0.0001, respectively.

We next examined glucocorticoid secretion by purines, pyrimidines, and their derived analogs. The basal glucocorticoid level (in non-treated H295R) was 40.1±4.147 pmol/10^4^ cells (Mean±SE, N = 6). As shown in Figure 2A, 2MeS-ATP, a P2Y_1_ agonist (100 µM and 1000 µM) significantly increased basal glucocorticoid levels to 64.62±3.54 (Mean±SE, N = 4; *p*<0.001) and 110.87±3.51 (Mean±SE, N = 4; *p*<0.0001) respectively, while none of the other purines, pyrimidines, or analogs known as purinergic agonists induced glucocorticoid secretion. A high concentration (100 µM) of adenosine (ADO) significantly reduced basal glucocorticoid levels to 23.11±0.89 (*p*<0.05).

We also placed an order with an analysis laboratory (SRL Co Ltd, Tokyo, Japan) to quantify basal, 1000 µM 2MeS-ATP-, 500 µM db-cAMP-, and 100 µM forskolin-induced cortisol secretion in the media with HPLC-RIA. The present fluorometric assay and HPLC-RIA were correlated well in the quantification of cortisol (Figure S1). The detection limit of fluorometric assay and HPLC-RIA was 0.02 and 0.01 (µg/mL), respectively (Table S1), and the interassay coefficient of variations (CVs) of the present method and HPLC-RIA were averaged 9.8% and 7.2%, respectively (Table S2 and S3).

### Extracellular Ca^2+^ and Glucocorticoid Secretion

Forskolin (100 µM), an activator of adenylyl cyclase, dibutyryl-cyclic AMP (db-cAMP), a permeable cAMP analog (250–500 µM), and angiotensin II (AngII, 100 nM) were used to confirm glucocorticoid secretion in H295R. All these agents induced glucocorticoid secretion from a basal level of 40.1±4.15 (pmol/10^4^ cells): forskolin (100 µM) to 126.4±9.15; db-cAMP (250 µM) to 87.4±2.36, and AngII (100 nM), to 80.8±12.8, (Fig. 2B, 2C). A previous study reported that glucocorticoid can be induced by a cAMP rise caused by activation of adenylyl cyclase, and also by penetration of db-cAMP to the cytosol from the extracellular environment [21]. However, in the present study, AngII (100 nM), with no linkage to cAMP, also increased glucocorticoid secretion to 80.8±12.8 pmol/10^4^ cells (Fig. 2B). Moreover, glucocorticoid secretion by db-cAMP was dependent on the extracellular Ca^2+^ concentration (Fig. 2C). In 1.2 mM Ca^2+^, 250 µM and 500 µM db-cAMP significantly increased the basal level (40.1±4.15) to 87.4±2.36 (*p*<0.001) and 82.6+4.94 (*p*<0.001), respectively. In 0.3 mM Ca^2+^, 250 µM and 500 µM db-cAMP did not significantly increased the basal levels of glucocorticoid secretion (40.7±1.46 and 38.5±2.09, respectively). In 0.6 mM Ca^2+^, 250 µM and 500 µM db-cAMP did not significantly increase the basal levels of glucocorticoid secretion (46.6±1.24 and 55.1±1.71, respectively). In 2.4 mM Ca^2+^, 250 µM and 500 µM db-cAMP significantly increased the basal level of glucocorticoid secretion to 97.0±5.72 (*p*<0.001) and 94.5+7.03, (*p*<0.001), respectively. In 4.8 mM Ca^2+^, 250 µM and 500 µM db-cAMP significantly increased the basal level to 81.5±3.44 (*p*<0.001) and 93.3±7.73 (*p*<0.001), respectively. On the other hand, under Ca^2+^-free conditions, no significant increase in the basal level was observed. The significance in these differences was analyzed for each point with purine agonists vs base (no agonists) in Figure 2A, and each agent vs base (no agents) in Figure 2B. A two-way ANOVA showed that the effect of each extracellular Ca^2+^ concentration with db-cAMP shown in Figure 2C was significant (*p*<0.0001). Thus, extracellular Ca^2+^ is essential for glucocorticoid secretion in H295R.

### Ca^2+^-mobilization in H295R

**Figure 3:**
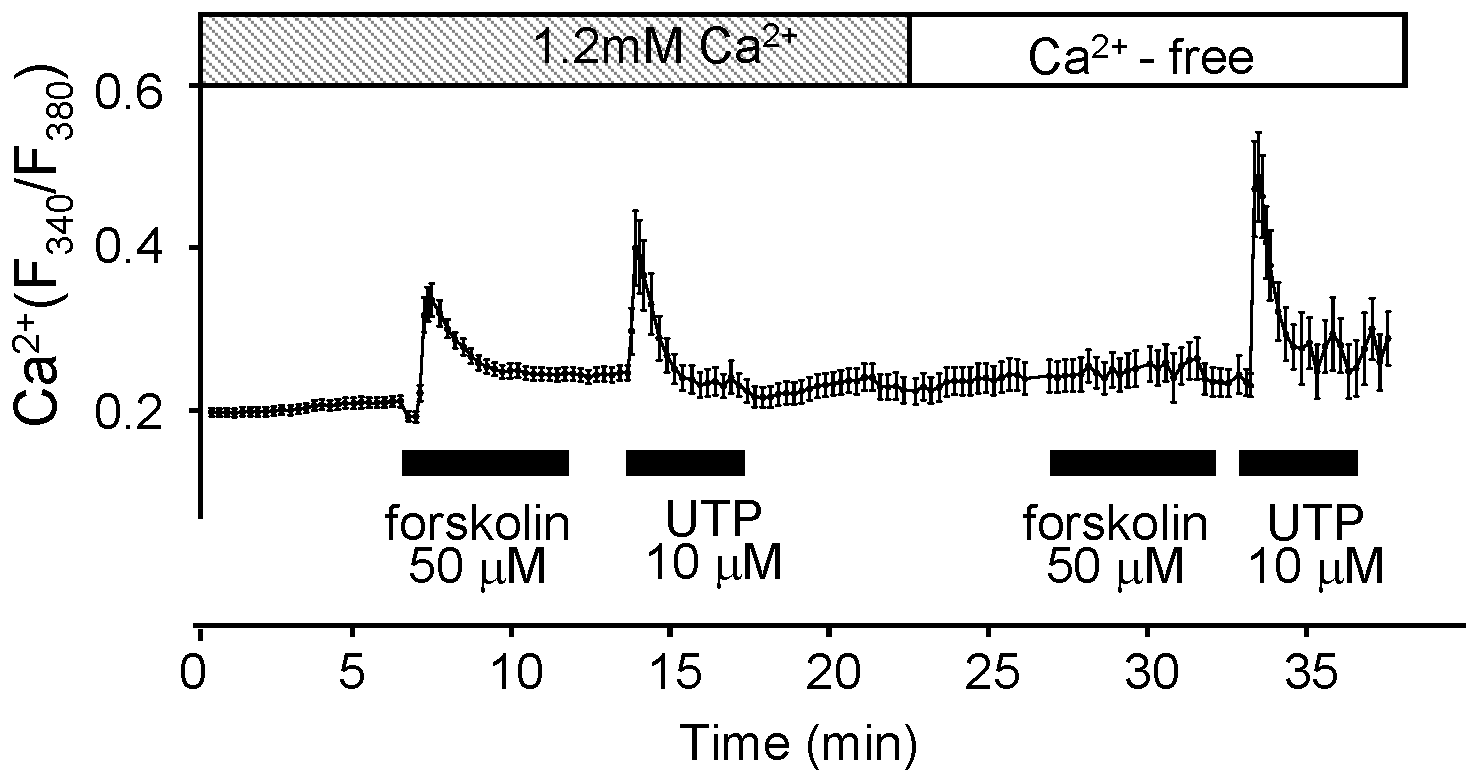
Ca^2+^-mobilization in H295R under 1.2 mM extracellular Ca^2+^ or Ca^2+^-free conditions. Shown is an averaged trace of the Ca^2+^-mobilization by forskolin and UTP in H295R under 1.2 mM extracellular Ca^2+^ or Ca^2+^-free conditions. The data were represented by the ratios of the fluorescent intensity (F_340_/F_380_). Each agonist was applied during the period indicated by solid bars. Each point with an error bar on the line represents the Mean±SD (N = 6).

We have previously reported that UTP, a P2Y_2_ agonist, induces Ca^2+^-mobilization and glucocorticoid secretion in BAFC, which express P2Y_2_ [20]. As shown in Figure 1, H295R also express this subtype of P2 receptor which is coupled to a G_q_ protein [4], [5], [6]. This means that activation of P2Y_2_ causes Ca^2+^ release from intracellular organelles independently of the existence of extracellular Ca^2+^. Next, we compared the effects of UTP and forskolin, an activator for adenylyl cyclase, causing cAMP rise without inducing Ca^2+^ release, on Ca^2+^-mobilization in H295R (Fig. 3). The intracellular Ca^2+^ concentration ([Ca^2+^]_i_) was represented as a ratio in individual cells, and the data were shown as the typical Ca^2+^-traces with standard deviations (SD) to indicate their variability. The Ca^2+^ peaks increased from the basal ratio with each stimulant.

Some differences in the elimination half lives of the peak ratios (the time constants: T_1/2_ =  τ) were observed in the Ca^2+^ trace of each nucleotide (Fig. 3). UTP enhanced a transient Ca^2+^ rise in both the presence and the absence of extracellular Ca^2+^, while forskolin induced Ca^2+^-mobilization only when extracellular Ca^2+^ (1.2 mM) was present. These results indicate that Ca^2+^-mobilization caused by UTP was due to intracellular Ca^2+^ release, not to Ca^2+^ influx from an extracellular site.

### Comparative assay of Ca^2+^-mobilization by Purinergic Agonists

**Figure 4:**
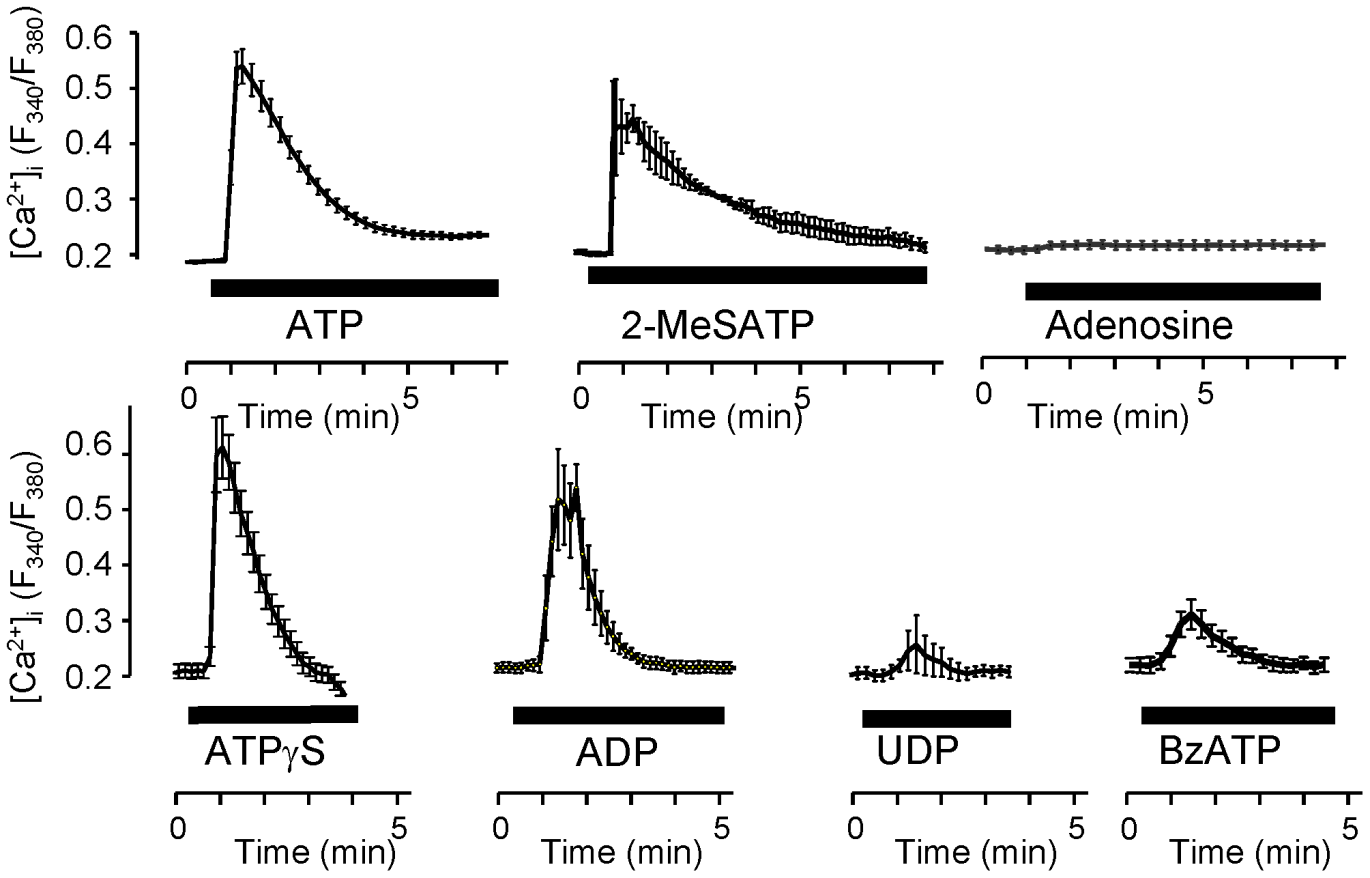
The effects of purine/pyrimidine agonists on transient Ca^2+^ in H295R. Shown is an averaged trace of the Ca^2+^-mobilization by nucleotides and nucleoside in H295R under 1.2 mM extracellular Ca^2+^ conditions. Adenosine did not induce a Ca^2+^ peak. Each purine agonist was applied at the concentration of 100 µM. Each point with an error bar on the line represents the Mean±SD (N = 6).

We examined the effect of purinergic agonists for both P1 and P2 on Ca^2+^-mobilization in H295R (Fig. 4). ATP, an agonist for P2X_5_, P2X_7_, and P2Y_2_, which does not produce glucocorticoid (Fig. 1), induced transient Ca^2+^-mobilization (Fig. 4). 2MeS-ATP, a P2Y_1_ agonist, which produces glucocorticoid (Fig. 1) also induced Ca^2+^-mobilization (Fig. 4). Adenosine, a P1 agonist, did not induce Ca^2+^-mobilization (Fig. 4). Adenosine 5′-gamma- thiotriphosphate (ATPγS), a potent P2Y_2_ agonist for BAFC activation [9], [22], ADP, another P2Y_1_ agonist, UDP, a P2Y_6_ agonist, and BzATP, a potent P2X_7_ agonist, induced Ca^2+^-mobilization (Fig. 4). 2MeS-ATP induced the slowest (Ca^2+^) decay of all the purine agonists (Fig. 4). The elimination half lives of the peak ratio (T_1/2_ =  τ) of ATP and 2MeS-ATP were 1.28±0.22 min and 2.42±0.14 min, respectively (Mean±SD, N = 6). No τ of the other purine agonists exceeded 1.00 min: ATPγS, 0.79±0.26; ADP, 0.66±0.28; UDP, 0.31±0.16; and BzATP, 0.70±0.21, (min, Mean±SD: N ≥6). Adenosine did not induce a Ca^2+^ peak (Fig. 4). All purine agonists were applied at a concentration of 100 µM.

### Ca^2+^-mobilization by AngII and db-cAMP

**Figure 5:**
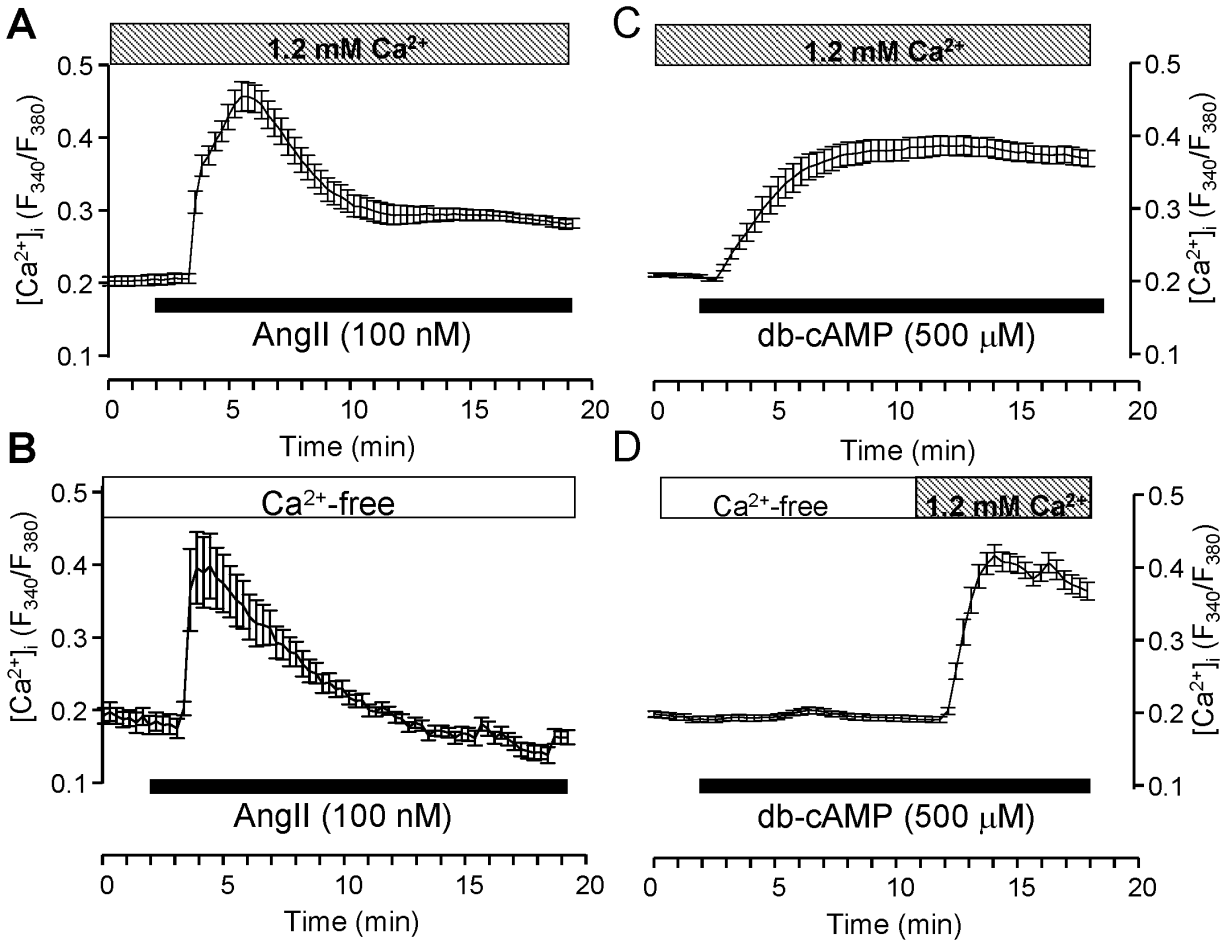
Averaged traces of the Ca^2+^-mobilization by AngII and db-cAMP in H295R. Each point with an error bar represents the Mean±SD (N = 5). Each agonist was applied during the period indicated by solid bars. White and lined columns indicate free of extracellular Ca^2+^ and 1.2 mM Ca^2+^ conditions, respectively.

AngII is known to act on AT_1_ receptors expressed in BAFC [10]. AT_1_ is also known to be coupled to G_q_ protein activation, resulting in Ca^2+^ release from intracellular stores [23], [24]. We have shown in Figure 2C that db-cAMP-induced glucocorticoid secretion was dependent on extracellular Ca^2+^. We next compared the changes in intracellular Ca^2+^ levels in H295R induced by AngII and db-cAMP in both the presence and the absence of extracellular Ca^2+^. AngII (100 nM) induced a transient peak of Ca^2+^-mobilization followed by a steady state which was significantly (*p*<0.05) higher than the basal level (Fig. 5A). The latter was abolished in the absence of extracellular Ca^2+^ (Fig. 5B). These results suggest that voltage activated Ca^2+^ channels and/or store-operated Ca^2+^ entry (SOCE) works in H295R. On the other hand, db-cAMP produced a slower Ca^2+^-mobilization in the presence of extracellular Ca^2+^ (Fig. 5C). No Ca^2+^-mobilization by db-cAMP was shown in the absence of extracellular Ca^2+^; however, extracellular Ca^2+^ (1.2 mM) alone produced a quick rise in [Ca^2+^]_i_ (Fig. 5D). The peak ratios of fluorescent intensity (F_340_/F_380_) and the τ by the agents represented in Mean±SD were: AngII, 0.44±0.054 and 3.56±0.98 min (N = 7); db-cAMP, 0.42±0.098 (N = 7) and 1.64±0.43 min (N = 5), respectively.

### Ca^2+^-mobilization by 2MeS ATP in the Presence or Absence of Extracellular Ca^2+^

**Figure 6:**
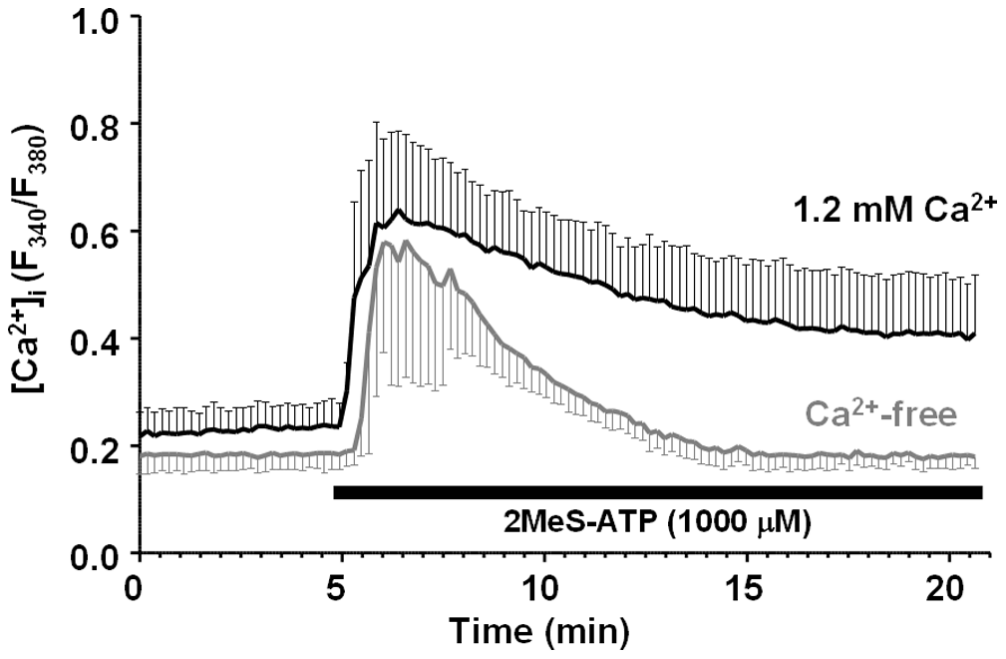
Comparative assay for the effects of extracellular Ca^2+^ on 2MeS-ATP-induced intracellular Ca^2+^-mobilization in H295R. A 1000 µM aliquot of 2MeS-ATP was applied to the cells under the extracellular Ca^2+^ (1.2 mM Ca^2+^) existence or Ca^2+^-free (with 2 mM EGTA) conditions. The peak ratios of the fluorescent intensity (F_340_/F_380_) and the τ by the agonist represented in Mean±SD (N = 14) were: extracellular 1.2 mM Ca^2+^, 0.64±0.123 and 7.42±2.78 min; extracellular Ca^2+^-free, 0.58±0.164 and 2.82±1.09 min, respectively. The difference between the τ from the two conditions was significant (*p*<0.001).

Comparative assay for the effects of extracellular Ca^2+^ on 2MeS-ATP-induced intracellular Ca^2+^-mobilization in H295R was done (Fig. 6). A 1000 µM aliquot of 2MeS-ATP was applied to the cells in the presence of extracellular Ca^2+^ (1.2 mM Ca^2+^) existence or Ca^2+^-free (with 2 mM EGTA) conditions. The peak ratios of the fluorescent intensity (F_340_/F_380_) and the τ by the agonist in Mean±SD (N = 14) were: in the presence of Ca^2+^, 0.64±0.123 and 7.42±2.78 min; in the absence of Ca^2+^, 0.58±0.164 and 2.82±1.09 min, respectively. The difference between the τ from the two conditions was significant (*p*<0.001).

### Analysis of SOCE in H295R

We examined whether extracellular purines induce store-operated calcium entry (SOCE) in H295R in the same way as described in Kawamura et al [25]. Thapsigargin (TG), a calcium ATPase inhibitor of endoplasmic reticulum (ER), was used for Ca^2+^ depletion of ER. In the present study, TG (2 µM) significantly increased the Ca^2+^ free basal ratio from 0.14±0.007 to 0.18±0.011 (*p*<0.001) transiently (τ = 4.23±0.46 min; N = 6).

**Figure 7:**
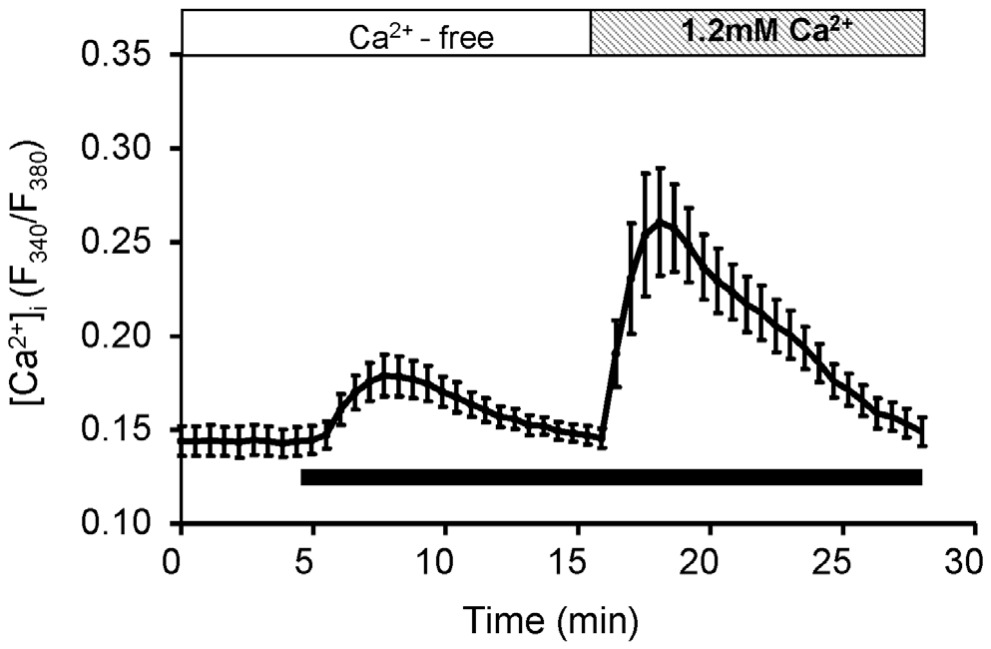
Averaged trace of the Ca^2+^-mobilization by thapsigargin in H295R. Each point with an error bar represents the Mean±SD (N = 6). Thapsigargin (TG), a calcium ATPase inhibitor of endoplasmic reticulum, was applied during the period indicated by solid bars. White and lined columns indicate free of extracellular Ca^2+^ and 1.2 mM Ca^2+^ conditions respectively. TG (2 µM) significantly increased the Ca^2+^-free basal ratio (0.14+0.007) to 0.18+0.011 (p<0.001) transiently. The 2nd Ca^2+^ peak was represented by the addition of 1.2 mM CaCl_2_ to the assay media.

A second Ca^2+^ increase (Ca^2+^ peak: 0.25±0.023; τ = 6.27±0.86 min) was caused by the addition of 1.2 mM CaCl_2_ to the assay media for a potent Ca^2+^ rise (Fig. 7).

### Detection of Key Molecules for SOCE in H295R

**Figure 8:**
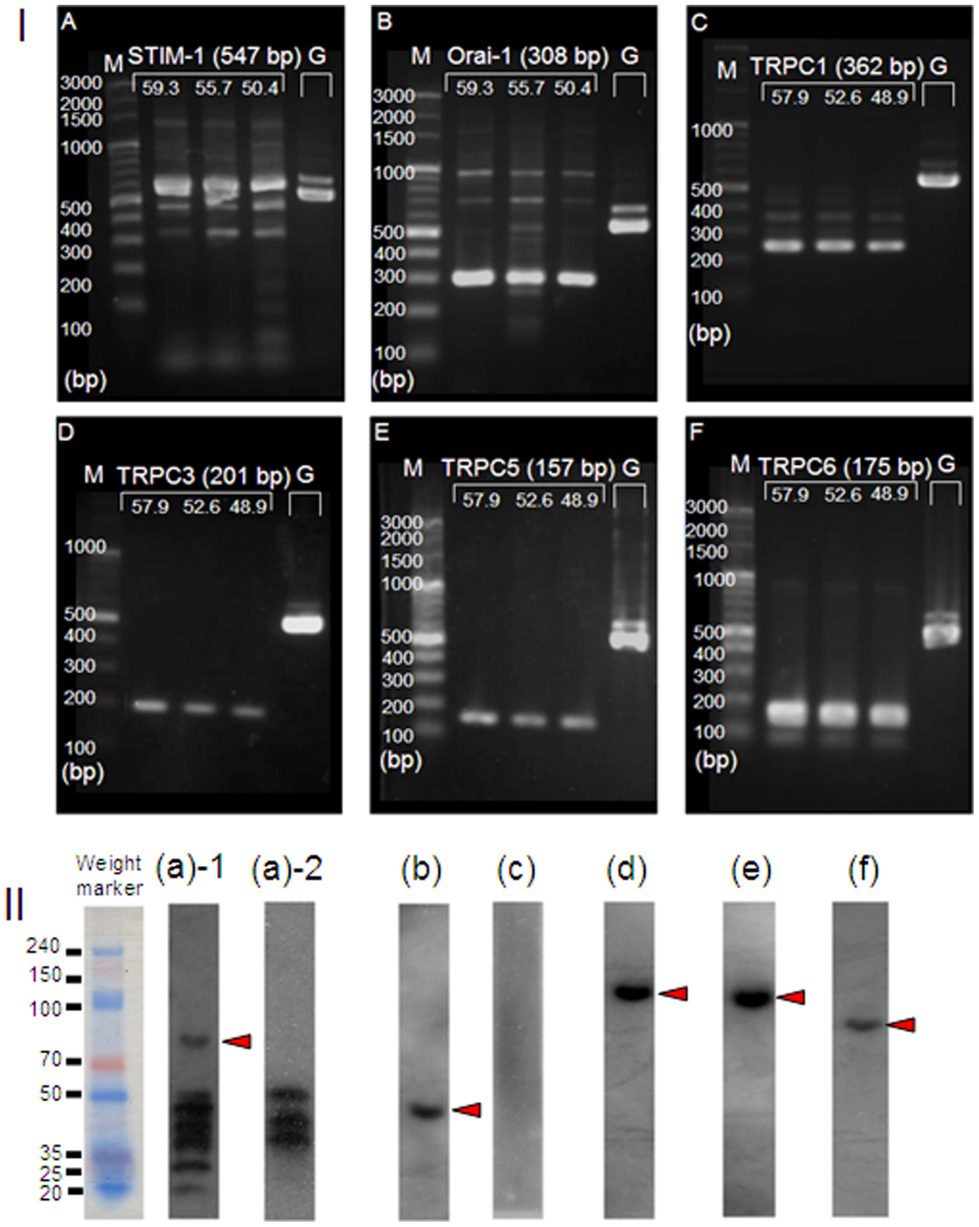
Detection of mRNA/protein for the key molecules for store operated calcium entry (SOCE) in H295R. I) mRNA, Stromal interaction molecule 1 (STIM1), a Ca^2+^ sensor expressed on endoplasmic reticulum, calcium release-activated calcium channel protein 1 (Orai-1), a component for connection between STIM1 and Ca channels, and TRPC channels, a possible Ca channel for SOCE were examined. mRNA for STIM1 (**A**) Orai-1 (**B**), and subtypes of TRPC1 (**C**), C3 (**D**), C5 (**E**), and C6 (**F**) were identified in H295R. The lanes in each gel image show: molecular markers (M), three PCR products for the mRNA target primers (products in high, middle, and low annealing temperatures are indicated), and one using a GAPDH primer (G). Each value in parentheses indicates the molecular weight (bp) for the expected PCR product. The numbers on the three lanes of the targets represent the annealing temperatures used in the individual procedures. II) protein, Anti-human antibodies for STIM1, Orai-1, and TRIPC channels were used to confirm the expression of proteins for which mRNA was detected by PCR in **figure 8**–**I**. The followings are the target proteins and their predicted molecular weights; **a**–**1:** STIM1 (84 kDa), **a**–**2:** STIM1 with control peptide (0.2 µg/mL) in the primary antibody reaction, **b:** Orai-1 (52 kDa), **c**: TRPC1 (83 kDa), **d:** TRPC3 (97 kDa), **e:** TRPC5 (110 kDa), **f:** TRPC6 (100 kDa). Molecular sizes (kDa), estimated by pre-stained weight marker, are shown on the left side of each membrane image. Each arrow head indicates the signal band that is clear and nearest to the predicted size of the target protein.

Stromal interaction molecule 1 (STIM1) and calcium release-activated calcium channel protein 1 (Orai-1) are known to be key molecules for SOCE. Recently, transient receptor potential cation channels C (TRPC) were reported to be necessary for the construction of SOC channels [26], [27]. In the present study, the expression of mRNA and protein for STIM1, Orai-1, and the C3, C5, and C6 subtypes of TRPC cannels (C2 is a pseudogene in humans) were identified in H295R (Fig. 8). In case of STIM-1, multiple bands were indicated (Fig. 8–II(a)–1). We used control peptides (NB110-60547PEP, Novus, Littleton, CO) which works competitively with anti-STIMI antibody (NB110-60547, Novus, Littleton, CO) to confirm the signal of STIM1. Some of the bands including the appropriate molecular band were disappeared (Fig. 8–II(a)–2).

### Detection of mRNA/Protein for Nucleotidases

**Figure 9:**
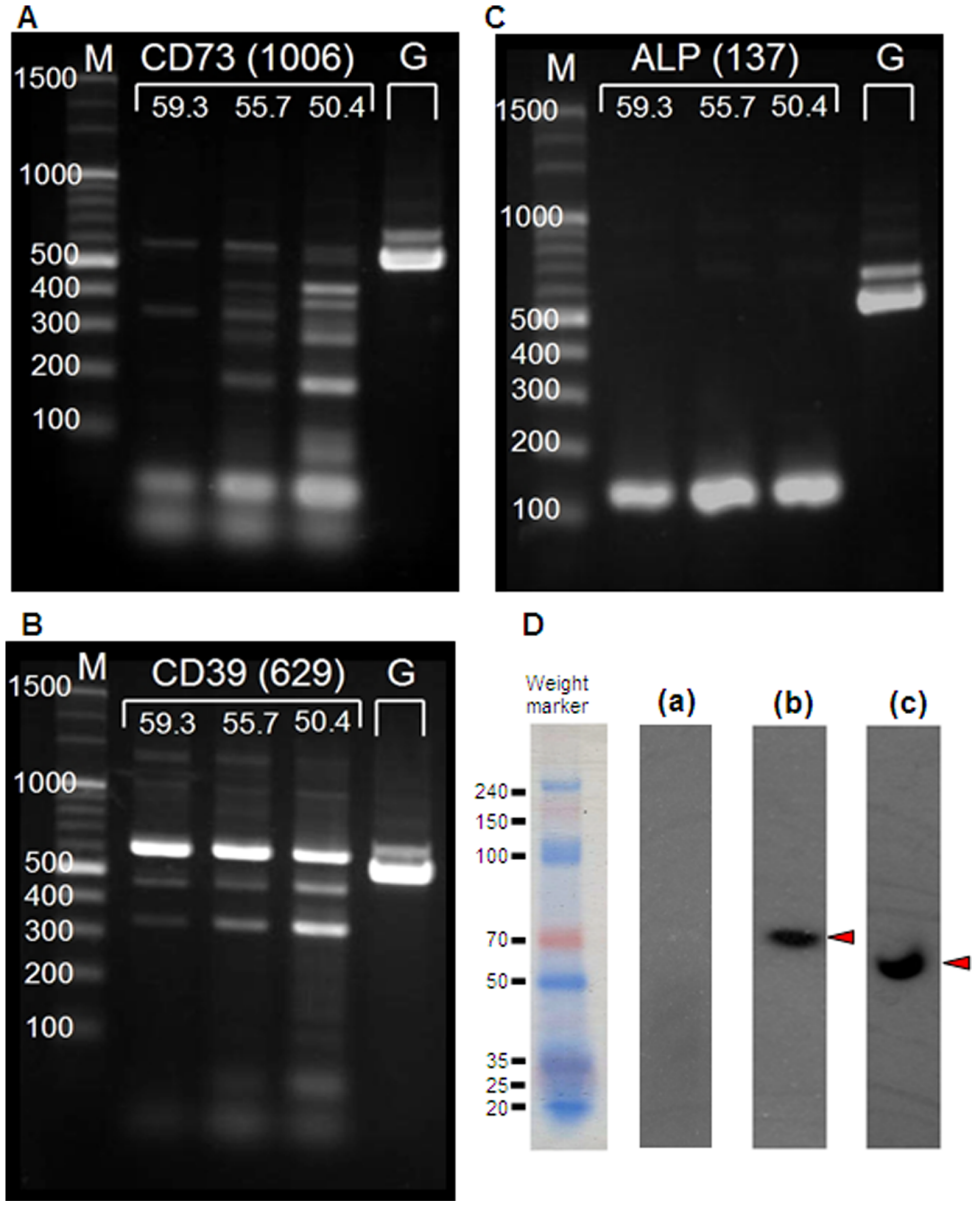
Detection of mRNA/protein for nucleotidases. Each image in A–C shows the PCR product bands at three different annealing temperatures, depending upon the melting temperatures (Tm) of the primers. The PCR product amplified for GAPDH (annealing temperature at 58°C) was also loaded on the same gel for each target. mRNA for ALP (**A**) and CD39 (**B**) were identified but CD73(**C**) was not. Furthermore, anti-human antibodies for ALP (**D**–**a**), CD39 (**D**–**b**), and CD73 (**D**–**c**) were used to confirm the expression of proteins for which mRNA was detected (CD39 and ALP) and not detected (CD73) by PCR shown in **A**–**C**. The signal on the PVDF membrane was indicated by the developing color (**D**). The followings are the target proteins and their predicted molecular weights; D–**a:** CD73 (29 kDa), D–**b:** CD39 (78 kDa), D–**c**: ALP (59 kDa). Molecular sizes (kDa), estimated by pre-stained weight marker, are shown on the left side of each membrane image. Each arrow head indicates the signal band that is clear and nearest to the predicted size of the target protein.

Each image in Figure 9A–C shows the PCR product bands at three different annealing temperatures, depending upon the melting temperatures (Tm) of the primers. The PCR product amplified for GAPDH (annealing temperature at 58°C) was also loaded on the same gel for each target. mRNA for CD39 (Fig. 9B) and ALP (Fig. 9C) were identified but CD73 (Fig. 9A) was not. Furthermore, anti-human antibodies for CD39 (Fig. 9D–b), ALP (Fig. 9D–c), and CD73 (Fig. 9D–a) were used to confirm the expression of proteins for which mRNA was detected (CD39 and ALP) or not detected (CD73) by PCR shown in figure 9A–9C.

### Metabolism Assay for ATP (I)

**Figure 10:**
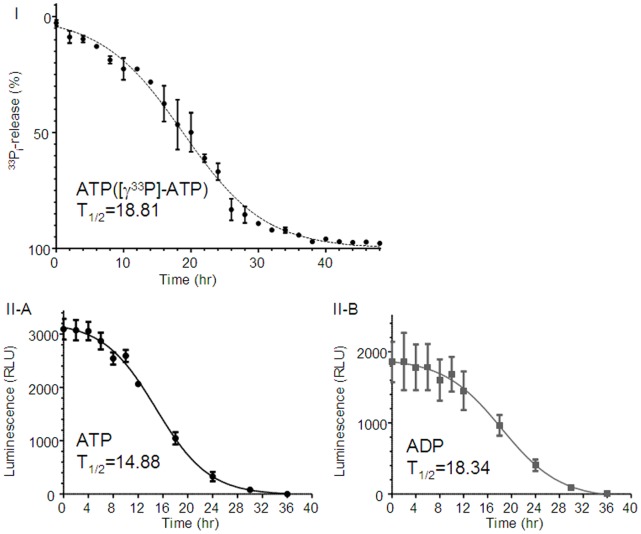
Metabolism assay for ATP and ADP. I) Metabolism assay for ATP (radio isotope assay). Ecto-ATP activity of H295R was determined by measuring the amount of [^33^P]-P_i_ released from [γ-^33^P]-ATP. H295R cultivated on 24-well plates were incubated in 1 mL of culture medium to which was added 1 mM ATP containing [γ-^33^P]-ATP (3.3 µM: 3.0 µCi/mL) for 48 hr. During the incubation, 5 µL of the medium was collected every 2 hr. The collected medium was added into 0.5 mL of PBS containing 50 µg of HCl-activated charcoal (Nacarai, Kyoto, Japan), then centrifuged in spin-column at 12,000×g for 1 min. Radioactivity in the extraction (400 µL) was counted using a beta counter. The amount of released [^33^P]-P_i_ was expressed as a percentage of the total [γ-^33^P]-ATP in 5 µL of the medium. ATP metabolism was then estimated. **II) Metabolism assay for ATP and ADP (luciferin-luciferase assay).** The graphs represent metabolism of ATP (**A**) and ADP (**B**). Data was plotted as the relative light units (RLU). Cells were exposed to either 1 mM ATP or 1 mM ADP and incubated for 48 hr. During the incubation, media was collected several times and assayed for ATP or ADP. Data represent the Mean±SE (N = 4). The curve fitting in each graph was performed by the GraphPad Prism (GraphPad Software, La Jolla, CA).

Ecto-ATP activity of H295R was determined by measuring the amount of [^33^P]-P_i_ released from [γ-^33^P]-ATP (Fig. 10–I). H295R cultivated on 24-well plates were incubated in 1 mL of culture medium to which was added 1 mM ATP containing [γ-^33^P]-ATP (3.3 µM: 3.0 µCi/mL) for 48 hr. During the incubation, 5 µL of the medium was collected every 2 hr. The collected medium was added into 0.5 mL of PBS containing 50 µg of HCl-activated charcoal (Nacarai, Kyoto, Japan), then centrifuged in spin-column at 12,000×g for 1 min. Radioactivity in the extraction (400 µL) was counting using a beta counter. The amount of released [^33^P]-P_i_ was expressed as a percentage of the total [γ-^33^P]-ATP in 5 µL of the medium. ATP metabolism was then estimated.

### Metabolism Assay for ATP and ADP (II)

The graphs in Figure 10–II represent metabolism of ATP (Fig. 10–II–A) and ADP (Fig. 10–II–B). Data was plotted as the relative light units (RLU). Cells were exposed to either 1 mM ATP or 1 mM ADP and incubated for 48 hr. During the incubation, media was collected several times and assayed for ATP or ADP. Data represent the Mean±SE (N = 4). The curve fitting in each graph was performed by the GraphPad Prism (GraphPad Software, La Jolla, CA).

### RNA Interference for P2Y_1_ mRNA

**Figure 11:**
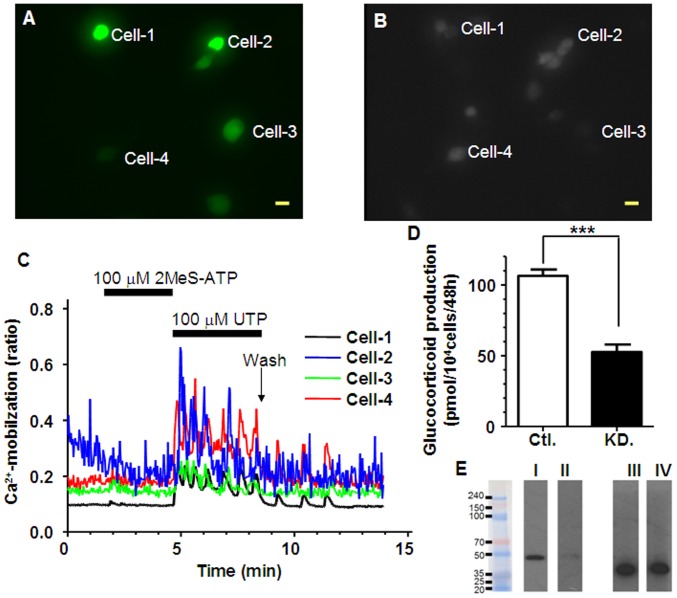
Effects of RNAi for P2Y_1_ mRNA on Ca^2+^-mobilization and GC secretion in H295R. shRNA plasmid-transfected H295R were cultured on coverslips or in 24-well plates for 48 hr. The cells were also loaded with fura2-AM for calcium assay. **A:** Image of eGFP signal-expressing H295R cultured on coverslips (excitation: 488 nm and elimination: 510 nm), **B:** The same field image as (**A**) but under the (excitation: 380 nm and the elimination at 510 nm. **C:** [Ca^2+^]_i_ traces in cell-1 to cell-4 indicated in (**A**) and (**B**). Each scale bar indicates 50 µm. **D:** Effect of shRNA transfection on 2MeS-ATP-induced glucocorticoid secretion in H295R. Cells were co-transfected with both the shRNA plasmid for P2Y_1_ and the eGFP plasmid (KD.). In control H295R (Ctl.), only the eGFP plasmid was transfected. GC secretion by 2MeS-ATP (106.4±5.27) was reduced in shRNA/eGFP-transfected H295R (52.6±6.49). Data represent the Mean±SE (N = 4). The ‘***’ indicates statistical significance at *p*<0.001. **E:** Western blotting analysis. **I:** P2Y_1_ in Ctl., **II:** P2Y_1_ in KD., **III:** GAPDH in Ctl., **IV:** GAPDH in KD.

RNA interference for P2Y_1_ mRNA was employed to show its importance as a target of 2MeS-ATP. In shRNA/eGFP-transfected H295R (Fig. 11A, 11B), UTP (100 µM) induced Ca^2+^-mobilization while 2MeS-ATP (100 µM) did not (Fig. 11C). GC secretion by 2MeS-ATP (106.4±5.27) was reduced in shRNA/eGFP-transfected H295R (52.6±6.49) but not in the eGFP-transfected H295R (control, Fig. 11D). Data represent the Mean±SE (N = 4).

## Discussion

The present study has shown, for the first time, that multiple purinergic receptors are expressed and some of them are linked to Ca^2+^-movilization/influx and cortisol secretion in H295R. The present findings have also shown that 2MeS-ATP, as one of the P2Y_1_-sensitive agonist, is the most effective purinergic agonist for glucocorticoid secretion. Furthermore, the present study has demonstrated that Ca^2+^ influx is critical for the (2MeS-ATP-induced) glucocorticoid secretion, and the present data indicate the possible involvement of SOCE activation.

H295R used in the present study are known as a pluripotent model that has all the key enzymes for steroidogenesis in the three layers of the adrenal cortex (zona glomerulosa, zona fasciculata, and zona reticularis), and can produce aldosterone, cortisol, and DHEA [11], [12], [13].

H295R expressed multiple purinergic receptors including G_s_-coupled P1 receptor and G_q/11_- or G_i/12_-coupled P2 receptors (Fig. 1). A_2A_ and A_2B_ are coupled to Gs protein; P2Y_1_, P2Y_2_, and P2Y_6_ are coupled to G_q/11_; P2Y_12_, P2Y_13_, and P2Y_14_ are coupled to G_i_; on the other hand, P2X_5_ and P2X_7_ are constructed as ligand-gated ion channels [1], [2]. Such a multiple expression of purinergic receptors in H295R could lead to activation of various intracellular effectors in the cells.

There are few agonists or antagonists that have absolute specificity for each subtype of purinergic receptors [2], [28]. The lack of specific ligands could prevent a robust pharmacological analysis of purinergic systems. We employed RNA interference procedure of P2Y_1_ mRNA, then identified that 2MeS-ATP activates Ca^2+^-mobilization and glucocorticoid secretion via P2Y_1_ (Fig. 11). These findings suggest that silencing/knockdown of the receptors might be an effective way for further investigation of the purinergic systems involved in Ca^2+^-mobilization and glucocorticoid secretion in H295R.

Among the multiple purinergic receptor agonists studied, only 2MeSATP showed a strong effect on glucocorticoid secretion, suggesting that P2Y_1_ is the only purinergic receptor linked to glucocorticoid secretion in H295R. On the other hand, ADP, another P2Y_1_ agonist, did not show any effect on glucocorticoid secretion in H295R. One possible explanation might be related to P2Y_13_ expression. ADP is also an agonist for P2Y_13_ [2], [28], and P2Y_13_ couples to a G_i_ protein that down-regulates cAMP. Up-regulation of cAMP is well known to lead to glucocorticoid secretion in adrenocortical cells [29]. This down-regulation of cAMP by ADP via P2Y_13_ may have some competitive effects on glucocorticoid secretion. In contrast to the present study, BAFC express only one type of purinergic receptor, a P2Y_2_ [20]. It is not clear whether the different expression is due to species difference.

One conceivable explanation for the ineffective stimulation of purine analogs might be related to the metabolization of purine analogs by nucleoside triphosphate diphosphohydrolases (NTPDases/CD39), ecto-5′-nucleotidase CD73 nucleotidase, or ecto-alkaline phosphatase [30]. It is reasonable that 2MeS-ATP worked well on glucocorticoid secretion in H295R, since it is a not-so-hydrolysable ATP analog [1]. In the present study, there was neither definite CD73 mRNA amplification (Fig. 9C) nor clear protein expression (Fig. 9D–c). These results suggest that some ecto-enzymes for nucleotides are indeed expressed in H295R. We also confirmed the lack of expression of “ecto”-ALP on their cell surface of H295R (Figure S2 and Text S1).

In order to check the activity of ecto-enzymes for ATP and/or ADP, we measured the alternation of ATP or ADP levels (due to metabolization) in the incubation media of H295R. Their metabolizations were observed but the half lives of ATP (Fig. 10–I, 10–II–A) or ADP (Fig. 10–II–B) were shown to be more than 14 to 18 hr, indicating that these slow metabolizations of nucleotides were caused by some other enzymes.

Stimulation by 2MeS-ATP induced at least a half efficacy in glucocorticoid secretion after a 12 hr-incubation (Figure S3). However, even 24 hr from the beginning of exposure to ATP or ADP, concentrations of these nucleotides remained above 100 µM (Fig. 10). This concentration level is thought to be efficient for activating the usual purinergic receptors [1], [2], [3]. However, as shown in Figure S3B, continuous exposure of 2MeS-ATP for 12 hr (followed by another 36 hr-incubation without 2MeS-ATP until 48 hr in total) was not very efficient. Even ATPγS, another slowly hydrolysable purine analog [22], [31], had no effect on glucocorticoid secretion in the cells (Fig. 2). These results suggest that not only ecto-enzyme activities (metabolization) that alter the applied purine agonists (e.g., dephosphorising ATP to ADP or ADO), but also other elements may work to control glucocorticoid secretion via purinergic systems. The present results also suggest that Ca^2+^ influx from the extracellular environment through P2X channels is insufficient for glucocorticoid secretion in H295R. The intracellular mechanisms between purinergic receptors and glucocorticoid secretion need to be further investigated.

Previous studies have reported that 2MeS-ATP activates P2X_5_ in myenteric neurons [32] and recombinant P2X_5_ transfected into HEK293 cells [33]. In the central nervous system, extracellular purines released from presynaptic nerve terminals act as “fast” neurotransmitters acting on ion channel-gated P2X receptors expressed postsynaptically to open the gates for extracellular Ca^2+^ [2], [28]. On the other hand, metabotropic GPCR types (P1 or P2Y) are involved in the “slow” transmission seen in hormone- or autacoid-like reactions [3]. Basically, the steroidogenesis involved in glucocorticoid secretion is a “slow” reaction [29], [34], and the only purinergic receptor identified in the present study that works for glucocorticoid secretion was P2Y_1_. It is well known that GPCR activation induces a transient [Ca^2+^]_i_ rise, which is independent of extracellular Ca^2+^ and is caused by intracellular inositol 1,4,5-triphosphate (InsP_3_) production followed by Ca^2+^ release from Ca^2+^ stores [35]. It has been also reported that P2Y_2_ activation by ATP or UTP induces InsP_3_ production and transient [Ca^2+^]_i_ rise independent of extracellular Ca^2+^ in BAFC [20]. On the other hand, activation of ion channel-gated P2X types does not induce a [Ca^2+^]_i_ rise under Ca^2+^-free conditions [36]. 2MeS-ATP-induced Ca^2+^-mobilization in the present study was shown in both the absence and the presence (1.2 mM) of Ca^2+^, suggesting that this agonist worked via P2Y_2_, not P2X_5_, in H295R.

Angiotensin II (AngII) is known to induce glucocorticoid secretion in BAFC [10] and H295R [37], [38]. In the present study, AngII induced both glucocorticoid secretion and Ca^2+^-mobilization with a Ca^2+^ peak, followed by a lasting phase (higher Ca^2+^ concentration than the base) in the presence of 1.2 mM Ca^2+^ (Fig. 5A). Because the lasting phase disappeared in the absence of Ca^2+^ (Fig. 5B), this may represent Ca^2+^ influx. In addition, AngII (and also 2MeS-ATP) did not induce glucocorticoid secretion in the absence of Ca^2+^ in H295R (Figure S4), further suggesting the essential role of Ca^2+^ influx.

UTP induced Ca^2+^ release but no Ca^2+^ influx (Fig. 4). The lack of sustained Ca^2+^ response may account for the lack of increased glucocorticoid secretion suggesting that Ca^2+^ influx is critical for glucocorticoid secretion in H295R. Indeed, ACTH-induced (cAMP-mediated) Ca^2+^ influx in glomerulosa cells was described by Gallo-Payet et al [19]. It was also reported by Spät [38] that extracellular Ca^2+^ is required for the sustained phase of ACTH-induced steroid secretion. Bird et al. [13] reported the Ca^2+^ dependence of 17 alpha-hydroxylase in H295R. In the case of adrenocortical cells, elevation of cAMP can open voltage dependent Ca^2+^ channels (VDCC) resulting in Ca^2+^-influx [39], [40], [41]. Furthermore it has been reported that AngII opens L-type [42], [43], T-type [44], [45], or N-type [46] VDCC. They could also contribute to Ca^2+^ influx in H295R.

Although, in the present study, L-type blocker did not inhibit 2MeS-ATP-induced glucocorticoid secretion but the blocker did reduce glucocorticoid secretion induced by AngII (Figure S5), the contribution of VDCC including N- or T-type channels could not be excluded and further investigation and further investigation is necessary.

The present study has also shown the expression of several mRNAs and proteins for the components of SOCE. The expression of multiple Ca^2+^ pathways in H295R [44], [45], [46] suggests that this cell has definite pathways that lead to activating distinct intracellular systems. Or, it might be based on the multiple steroidogenic activity of H295R [12], [34]. In the present study, we observed “plateau”-like phases linked to an increase in the “τ” in Ca^2+^-mobilization.

In conclusion, the present study has shown for the first time that H295R express multiple P2 purinergic receptors. Activations of some of them lead to receptor-activated Ca^2+^-mobilizations (release and influx), and the Ca^2+^ influx is critical for glucocorticoid secretion. These findings also suggest that P2Y_1_ may be linked to glucocorticoid secretion via SOCE in H295R. Complex purinergic system linked to Ca^2+^ release and influx, including SOCE, may play an important role in steroidogenesis in the human adrenal cortex.

## Materials & Methods

### Chemicals

Dulbecco’s modified Eagle’s medium/Ham’s F-12 medium was obtained from GIBCO Industries, Inc. (Los Angeles, CA). Fetal bovine serum (FBS) was purchased from HyClone Laboratories (Logan, UT). Antibiotics (penicillin and streptomycin) were purchased from the Meiji Pharma Co. Ltd. (Tokyo, Japan). The ITS+ supplement (insulin, transferrin, selenium, and linoleic acid) was obtained from Collaborative Research (Bedford, MA). ATP, adenosine diphosphate (ADP), adenosine, uridine triphosphate (UTP), uridine diphosphate (UDP), and 2-(methylthio) adenosine 5′-triphosphate (2MeS-ATP) were purchased from Sigma-Aldrich, Inc. (St. Louis, MO). Adenosine-5′-O-(3-thiotriphosphate) (ATPγS) was obtained from Enzo Life Sciences, Inc. (Plymouth, PA). All other chemicals were of analytical grade.

### Cell Culture

NCI-H295R cells [12] were obtained from ATCC (American Type Culture Collection, Manassas, VA), and cultured in Dulbecco’s modified Eagle’s medium/Ham’s F-12 medium (1∶1, 15 mM HEPES) supplemented with: 2.5% fetal calf serum, and 1% ITS+ supplement (insulin, 6.25 µg/ml; transferrin, 6.25 µg/ml; selenium, 6.25 ng/ml; and linoleic acid, 5.35 µg/ml; Collaborative Research, Bedford, MA). All media contained 100 µg/ml streptomycin (Sigma, St. Louis, MO).

### RNA Isolation and RT-PCR

Total RNA was isolated; digestion of DNA fragments in the isolated RNA, and purification of the RNA was performed using Total RNA Isolation (Macherey-Nagel, Duren, Germany). Following spin column purification, RNA samples were reverse-transcripted to cDNA using the PrimeScript II 1st strand cDNA synthesis kit (Takara, Kyoto, Japan) with oligo-dT (Promega, Fitchburg, WI). The cDNA then underwent PCR using QuickTaq HS DyeMix (Toyobo, Osaka, Japan) for 33 cycles. PCR products were subjected to gel electrophoresis on a 2.0–2.5% agarose gel, depending on the expected molecular size of the PCR products. PCR conditions were identical for all primers (Table S4).

### PAGE-Western Blotting

H295R were lysed in RIPA Buffer (Thermo Scientifics, Rockford, IL) with protease inhibitor cocktail (Nacarai Tesuque, Kyoto, Japan). The crude lysate was treated to remove the extraction buffer with the Pierce SDS-PAGE Sample Kit (Thermo Scientifics, Rockford, IL). The extracted protein samples (2.8–3.3 mg protein/mL) were mixed with the same volume of the EzApply Sample Buffer (Atto, Tokyo, Japan) containing dithiothreitol (100 mM) and were heated at 98°C for 5 min (37°C for 15 min in some cases depending on the target protein). The heat-treated samples were applied to the Perfect NT Ge (5–20% gradient), a precast gel (DRC, Tokyo, Japan) for PAGE (200 V – 35 min). Following PAGE, the protein samples were transferred to the ClearBlot, a PVDF membrane (Atto, Tokyo, Japan) by Western blotting (400 mA – 60 min).

### Antibodies Binding

Membranes containing transferred proteins were treated with bovine serum albumin (5%) for 1 hr at room temperature. Following blocking, the PVDF membrane was cut into pieces along the locus lanes of the LandMark Broad-range Prestained Protein Marker (DRC, Tokyo, Japan), a pre-stained molecular marker. These rectangular membrane strips were treated with each primary antibody diluted in Solution-I of the CanGetSignal System (Toyobo, Kyoto, Japan), the immunoreaction enhancer solution, for 1 hr at room temperature. After washing with TBS-Tween (0.05–0.1%), the membrane pieces were reacted with 2ndary antibodies in Solution-II of the CanGetSignal System for 1 hr at room temperature. Washed membranes were then dumped into EzWetLumi plus Solution (Atto, Tokyo, Japan), a highly sensitive substrate for HRP, to detect the developing chemiluminescence signals for the HRP-conjugated 2ndary antibodies that bind to the primary antibodies on the PVDF membranes. X-ray films (Kodak, Rochester, NY) were exposed to the PVDF membranes, and were developed by a film developing system (SRX-101, Konica Minolta, Tokyo, Japan). The developed films were captured by a scanner system (GTX-970, Epson, Nagano, Japan) to detect the bands on the PVDF membranes. According to the explanation appendixes of the antibodies used, multiple signal bands were occasionally expected. In the case of multiple bands, the control peptide for the antibody was used for competitive inhibition. Supporting details are in the Text S1.

### Glucocorticoid Secretion Assay

The adherent cultured H295R were washed twice with Ca^2+^-free, phosphate buffered saline containing 0.5 mM EGTA (PBS-EGTA), followed by washing once with HamF12/DMEM medium. The washed cells were incubated at 37°C for appropriate hrs in a total incubation volume of 1 ml in a humidified atmosphere of 5% CO_2_ in air. At the end of the incubation, 0.5 ml of each incubation medium was removed for the corticosteroid assay. Then the cells were detached for a 10 min incubation period at 37°C in 0.1% trypsin containing PBS-EGTA to count the cell number by a hemocytometer. The removed media for assay was mixed with CH_2_Cl_2_ to extract corticosteroid. The corticosteroid in CH_2_Cl_2_ was transferred to the sulfuric acid reagent (H_2_SO_4_ : ethanol = 7∶ 3) and mixed well. The emission of 520 nm excited by 390 nm was measured to quantify corticosteroid using cortisol as a standard [34], [47].

### Intracellular Ca^2+^ Measurements

H295R cells that had been cultured for one day on cover slips treated for cell adhesion (Fisher Scientific, Hampton, NH), were used for intracellular Ca^2+^ concentration ([Ca^2+^]_i_) measurements. The cells were loaded with 5 µM fura-2 acetoxymethylester (fura-2/AM). The fluorescence was monitored at an emission wavelength of 510 nm by the imaging system operated by The AquaCosmos (Hamamatsu Photonics, Hamamatsu, Japan). The [Ca^2+^]_i_ was shown as the ratio of the 510 nm fluorescence intensity excited at 340 nm, to that at 380 nm.

### RNA Interference for P2Y_1_ mRNA

To generate the vector for short hairpin RNA (shRNA ) for use in RNA interference (RNAi), pENTR/U6 plasmid (Invitrogen, Carlsbad, CA) with a DNA cassette for shRNA was used. The cassette for shRNA was composed of double strand DNA. Each DNA strand was called either ‘top’ or ‘bottom’. We checked the sequences for RNAi using the program on Invitrogen’s site and selected the sequence of the target mRNA (P2Y_1_) for RNAi. A cassette for the target was made by annealing the top and the bottom, followed by cloning the double strand DNA into pENTR/U6 using T4 DNA ligase (Invitrogen, Carlsbad, CA). Finally we selected two different targets in P2Y_1_ mRNA and made two different cassettes. The top strand of cassette-1, the bottom strand of cassette-1, the top strand of cassette-2, and the bottom strand of cassette-2 were: CACCGCTGTCTACATCTTGGTATTCCGAAGAATACCAAGATGTAGACAGC, AAAAGCTGTCTACATCTTGGTATTCTTCGGAATACCAAGATGTAGACAGC, CACCGCCACGTATCAGGTGACAAGACGAATCTTGTCACCTGATACGTGGC, and AAAAGCCACGTATCAGGTGACAAGATTCGTCTTGTCACCTGATACGTGGC, respectively. A set of the top and the bottom strands of the DNA was made. The plasmid was transformed using the One Shot TOP10 Chemically Competent *E. coli* (Invitrogen, Carlsbad, CA) and incubated at 37°C in LB followed by spreading on an LB agar plate with kanamycin (50 µg/mL) at 37°C overnight. Some colonies were selected and amplified in LB with kanamycin. Amplified plasmids were extracted and purified to use in transfection. Transfection was done in suspended H295R by electroporation with electroporator MP-100 (NanoEnTek, Seoul, Korea) and eGFP plasmid was co-transfected with the shRNA plasmid. As a control, a plasmid for a non-coding cassette was used and eGFP plasmid was again co-transfected. Each plasmid (0.5 µg) was added to 10^5^ cells in 10 µL electrode buffer (Invitrogen, Carlsbad, CA) followed by pulsation (1800 V – 20 msec).

### Statistical Analyses

Values are expressed as Mean±SE or Mean±SD (in case of real time Ca^2+^ measurements) and data were analyzed by one- or two-way analysis of variance (ANOVA) followed by Tukey-Kramer post-hoc tests in order to reduce the false positive or type I error rate. A *p-*value of less than 0.05 was considered to be statistically significant.

## Supporting Information

Figure S1 **Correlation analysis between the fluorometric analysis and HPLC-RIA for cortisol quantifications.** Amounts of cortisol in the culture medium quantified by the fluorometric analysis correlate with the results obtained by HPLC-RIA (r = 0.9340). In these tests, basal levels, those stimulated by 1000 µM 2MeS-ATP, by 500 µM db-cAMP, and by 100 µM forskolin were compared between the fluorometric analysis and HPLC-RIA (N = 4–6).
(TIF)

Figure S2 **Analysis of expression of ecto-alkaline phosphatase.** Images of ALP (**1**) and TRPC5 (**2**) on the H295R cell surface. **1A** and **2A**: transparent images, **1B** and **2B**: images of Alexa 488 as protein expression, 1C and 2C: images of DAPI for nucleoli, and **1D** and **2D**: overlay of A, B, and C. For DyLight 488-labeled 2ndary antibody (Alexa Fluor 488) examination, cells were excited at 488 nm and the emission was observed through a 520 nm band path filter, respectively. For DAPI, the cells were excited at 358 nm and the emission was observed through a 460 nm band path filter. TRPC5 (**2B**) expression is positive but ALP is not (**1B**) as ecto-ALP. All images were originally observed as monochromes and changed to pseudo-colors. Scale bar indicate 50 µm.
(TIF)

Figure S3 **Time-dependent cortisol secretion assay in H295R. A) Time-course of cortisol secretion in H295R.** Time course study of cortisol secretion by 1 mM 2MeS-ATP in H295R. Data represent the Mean±SE (N = 4). The curve fitting in A was performed by the GraphPad Prism (GraphPad Software, La Jolla, CA). **B) Cortisol secretion following short-term incubation in H295R.** Short-term incubation procedures were performed using 2MeS-ATP. The media was removed from the well at 1, 2, 4, 8, 12, 24, or 36 hr after application of the agents. Once the media was removed, the wells were washed and refilled with the same volume (1 mL) of fresh media and maintained in incubation without any agents until 48 hr. Graph shows cortisol secretion at 48 hr after the application. Data represent the Mean±SE (N = 4).
(TIF)

Figure S4 **Effects of purinergic agonists on GC secretion under Ca^2+^-free conditions in H295R.** No agonists tested under Ca^2+^-free conditions (2 mM EGTA) induced significant GC secretion. For comparison, GC secretion by 2MeS-ATP under standard condition (1.2 mM Ca^2+^) is shown as Mean±SE (N = 4).
(TIF)

Figure S5 **Comparative assay for the effect of an L-type VDCC blocker on 2MeS-ATP- or AngII-induced glucocorticoid secretion.** Effects of nifedipine, an L-type VDCC-blocker, on 2MeS-ATP or AngII-induced glucocorticoid secretion in H295R. The cells were incubated at 37°C for 48h. Each histogram represents the Mean±SE (N = 4). ‘*’, ‘**’, statistical significance at *p*<0.05, *p*<0.01, respectively.
(TIF)

Table S1 **Sensitivity of cortisol assay in the fluorometric analysis and HPLC-RIA.**
(DOC)

Table S2 **Interassay coefficient of variations (CVs) in the fluorometric analysis for cortisol (N = 4–6).**
(DOC)

Table S3 **Interassay coefficient of variations (CVs) in HPLC-RIA for cortisol (N = 4).**
(DOC)

Table S4 **Gene specific primers used for PCR amplification.**
(DOC)

Text S1 **Supporting Information.**
(DOC)
