# Supplementary figures and images for: NCI-H295R, a Human Adrenal Cortex-Derived Cell Line, Expresses Purinergic Receptors Linked to Ca2+-Mobilization/Influx and Cortisol Secretion
Source: PLoS One. 2013 Aug 8;8(8):e71022. doi: 10.1371/journal.pone.0071022 (PMC3738630; doi:10.1371/journal.pone.0071022)

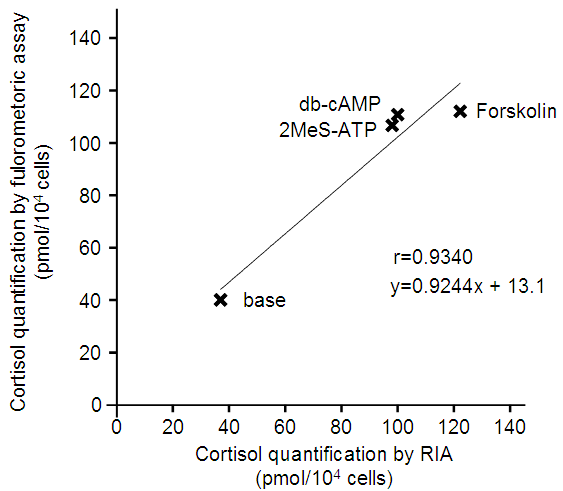

Supplement: Figure S1 — Correlation analysis between the fluorometric analysis and HPLC-RIA for cortisol quantifications. Amounts of cortisol in the culture medium quantified by the fluorometric analysis correlate with the results obtained by HPLC-RIA (r = 0.9340). In these tests, basal levels, those stimulated by 1000 µM 2MeS-ATP, by 500 µM db-cAMP, and by 100 µM forskolin were compared between the fluorometric analysis and HPLC-RIA (N = 4–6). (TIF) [file pone.0071022.s001.tif]

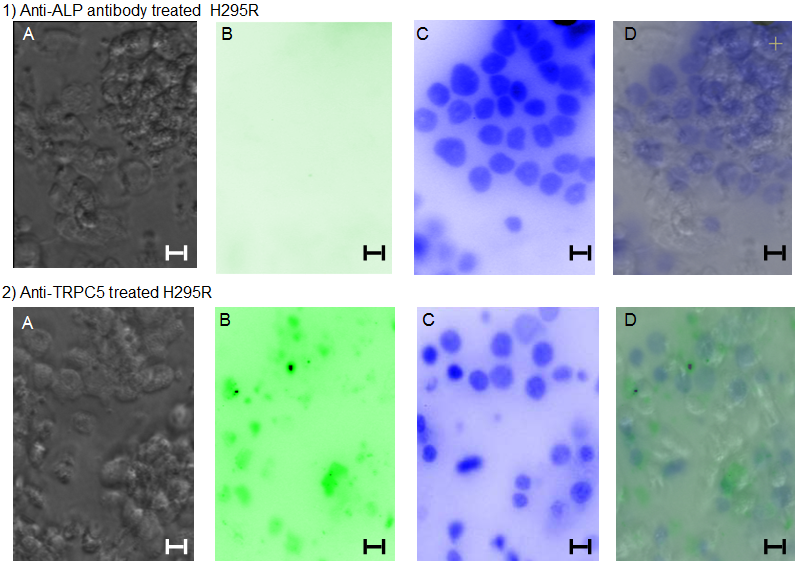

Supplement: Figure S2 — Analysis of expression of ecto-alkaline phosphatase. Images of ALP (1) and TRPC5 (2) on the H295R cell surface. 1A and 2A: transparent images, 1B and 2B: images of Alexa 488 as protein expression, 1C and 2C: images of DAPI for nucleoli, and 1D and 2D: overlay of A, B, and C. For DyLight 488-labeled 2ndary antibody (Alexa Fluor 488) examination, cells were excited at 488 nm and the emission was observed through a 520 nm band path filter, respectively. For DAPI, the cells were excited at 358 nm and the emission was observed through a 460 nm band path filter. TRPC5 (2B) expression is positive but ALP is not (1B) as ecto-ALP. All images were originally observed as monochromes and changed to pseudo-colors. Scale bar indicate 50 µm. (TIF) [file pone.0071022.s002.tif]

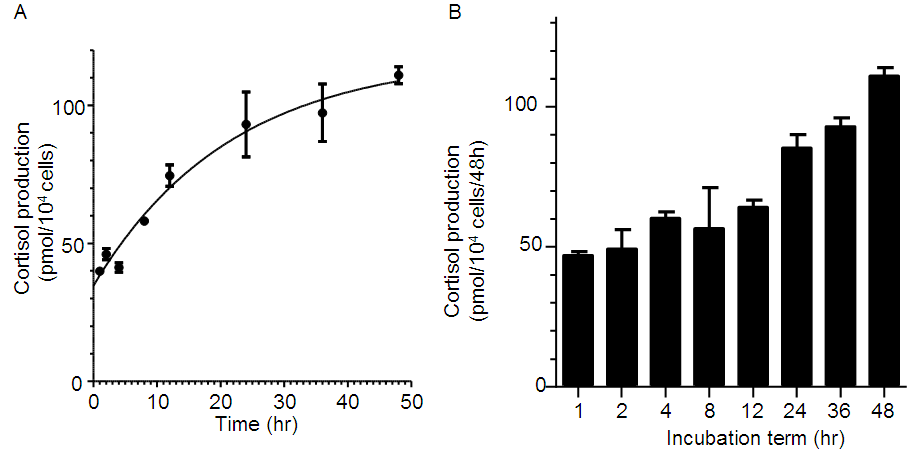

Supplement: Figure S3 — Time-dependent cortisol secretion assay in H295R. A) Time-course of cortisol secretion in H295R. Time course study of cortisol secretion by 1 mM 2MeS-ATP in H295R. Data represent the Mean±SE (N = 4). The curve fitting in A was performed by the GraphPad Prism (GraphPad Software, La Jolla, CA). B) Cortisol secretion following short-term incubation in H295R. Short-term incubation procedures were performed using 2MeS-ATP. The media was removed from the well at 1, 2, 4, 8, 12, 24, or 36 hr after application of the agents. Once the media was removed, the wells were washed and refilled with the same volume (1 mL) of fresh media and maintained in incubation without any agents until 48 hr. Graph shows cortisol secretion at 48 hr after the application. Data represent the Mean±SE (N = 4). (TIF) [file pone.0071022.s003.tif]

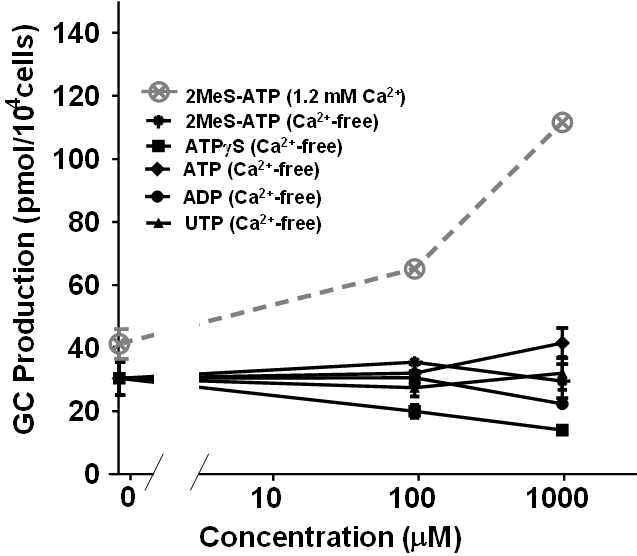

Supplement: Figure S4 — Effects of purinergic agonists on GC secretion under Ca2+-free conditions in H295R. No agonists tested under Ca2+-free conditions (2 mM EGTA) induced significant GC secretion. For comparison, GC secretion by 2MeS-ATP under standard condition (1.2 mM Ca2+) is shown as Mean±SE (N = 4). (TIF) [file pone.0071022.s004.tif]

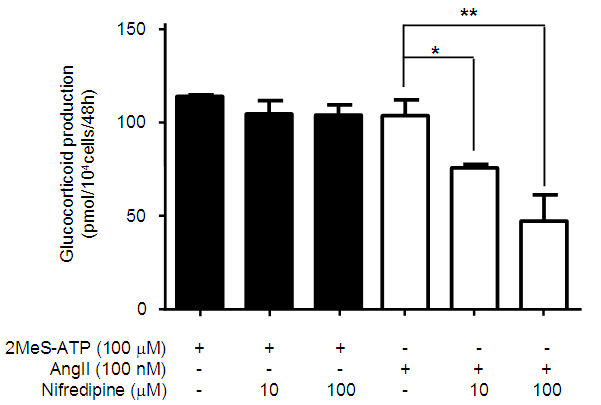

Supplement: Figure S5 — Comparative assay for the effect of an L-type VDCC blocker on 2MeS-ATP- or AngII-induced glucocorticoid secretion. Effects of nifedipine, an L-type VDCC-blocker, on 2MeS-ATP or AngII-induced glucocorticoid secretion in H295R. The cells were incubated at 37°C for 48h. Each histogram represents the Mean±SE (N = 4). ‘*’, ‘**’, statistical significance at p<0.05, p<0.01, respectively. (TIF) [file pone.0071022.s005.tif]
