# Supplementary material for: NCI-H295R, a Human Adrenal Cortex-Derived Cell Line, Expresses Purinergic Receptors Linked to Ca2+-Mobilization/Influx and Cortisol Secretion
Source: PLoS One. 2013 Aug 8;8(8):e71022. doi: 10.1371/journal.pone.0071022 (PMC3738630; doi:10.1371/journal.pone.0071022)
Supplement: Table S1 — Sensitivity of cortisol assay in the fluorometric analysis and HPLC-RIA. (DOC) [file pone.0071022.s006.doc]

Table S1. Sensitivity of cortisol assay in the fluorometric analysis and HPLC-RIA

| Cortisol assay | Detection limit (μg/mL) | Average of CV (%) |
| --- | --- | --- |
| Fluorometric analysis  RIA | 0.02*  0.01** | 9.8  7.1 |

* Based on detection limit of the present study, 10 ng/0.5 mL.

** Based on the system in the commission research lab (SRL Co Ltd).
