# Supplementary material for: NCI-H295R, a Human Adrenal Cortex-Derived Cell Line, Expresses Purinergic Receptors Linked to Ca2+-Mobilization/Influx and Cortisol Secretion
Source: PLoS One. 2013 Aug 8;8(8):e71022. doi: 10.1371/journal.pone.0071022 (PMC3738630; doi:10.1371/journal.pone.0071022)
Supplement: Table S2 — Interassay coefficient of variations (CVs) in the fluorometric analysis for cortisol (N = 4–6). (DOC) [file pone.0071022.s007.doc]

Table S2. Interassay coefficient of variations (CVs) in the fluorometric analysis for cortisol (N = 4-6).

| Stimulation | Mean (pmol/10^5^ cells) | SD | CV (%) |
| --- | --- | --- | --- |
| 2MeS-ATP (10 μM)  2MeS-ATP (100 μM)  2MeS-ATP (1000 μM)  BzATP (10 μM)  BzATP (100 μM)  BzATP (1000 μM)  UTP (10 μM)  UTP (100 μM)  UTP (1000 μM)  UDP (10 μM)  UDP (100 μM)  UDP (1000 μM)  ATP (10 μM)  ATP (100 μM)  ATP (1000 μM)  ADP (10 μM)  ADP (100 μM)  ADP (1000 μM)  ATPgS (10 μM)  2MeS-ATP (100 μM)  2MeS-ATP (1000 μM)  Adenosine (10 μM)  Adenosine (100 μM)  Adenosine (1000 μM)  db-cAMP (500 μM)  Forskolin (100 μM)  Ang II (100 nM)  Base | 47.9  64.6  110.8  51.6  52.5  43.6  37.2  40.7  41.5  38.7  38.1  39.7  41.4  43.1  42.3  46.4  41.1  32.5  42.2  43.3  40.2  42.1  43.3  23.3  106.7  112.1  80.7  40.1 | 0.85  6.14  6.08  10.6  6.17  0.25  4.84  6.33  1.96  2.11  3.36  3.33  1.09  3.02  3.38  7.9  1.64  2.69  14.24  3.81  10.21  1.41  5.73  1.59  1.22  5.59  11.03  4.41 | 1.8  9.5  5.5  19.4  11.8  0.6  13.0  15.6  4.7  5.5  8.8  8.4  2.6  7.0  9.1  17.0  4.0  8.3  33.7  8.8  25.4  3.3  13.2  6.8  1.1  5.0  13.7  10.1 |

Average of CV in the fluorometric analysis: 9.8%
