# Supplementary material for: NCI-H295R, a Human Adrenal Cortex-Derived Cell Line, Expresses Purinergic Receptors Linked to Ca2+-Mobilization/Influx and Cortisol Secretion
Source: PLoS One. 2013 Aug 8;8(8):e71022. doi: 10.1371/journal.pone.0071022 (PMC3738630; doi:10.1371/journal.pone.0071022)
Supplement: Table S3 — Interassay coefficient of variations (CVs) in HPLC-RIA for cortisol (N = 4). (DOC) [file pone.0071022.s008.doc]

Table S3. Interassay coefficient of variations (CVs) in HPLC-RIA for cortisol (N = 4).

| Stimulsation | Mean (pmol/10^5^ cells) | SD | CV (%) |
| --- | --- | --- | --- |
| 2MeS-ATP (1000 μM)  db-cAMP (500 μM)  Forskolin (100 μM)  Base (no-stimulation) | 98.3  100.0  122.2  37.9 | 7.94  7.45  5.79  3.00 | 8.1  7.4  4.75  7.9 |

Average of CV in HPLC-RIA : 7.1%
