# Supplementary material for: NCI-H295R, a Human Adrenal Cortex-Derived Cell Line, Expresses Purinergic Receptors Linked to Ca2+-Mobilization/Influx and Cortisol Secretion
Source: PLoS One. 2013 Aug 8;8(8):e71022. doi: 10.1371/journal.pone.0071022 (PMC3738630; doi:10.1371/journal.pone.0071022)
Supplement: Table S4 — Gene specific primers used for PCR amplification. (DOC) [file pone.0071022.s009.doc]

Table S4. For PCR amplification, the following gene specific primers were used:

| Targets | | Primer sequences | Tm*  (^o^C) | Size  (bp) |
| --- | --- | --- | --- | --- |
| A_1_ | Sense | CTCGCCATCCTCATCAACATT | 57.1 | 245 |
|  | Antisense | CAGCCAAACATAGGGGTCAGTC | 58.0 |  |
| A_2A_ | Sense | GCCCCTCTCTGGCTCATGTACCTG | 64.0 | 450 |
|  | Antisense | TCATCAGGACACTCCTGCTCCATC | 61.7 |  |
| A_2B_ | Sense | GAGCTGATGGACCACTCGAGG | 60.5 | 341 |
|  | Antisense | ACACCGAGAGCAGGCTGTACC | 61.1 |  |
| A_3_ | Sense | AACGTGCTGGTCATCTGCGTGGTC | 64.2 | 441 |
|  | Antisense | GTAGTCCATTCTCATGACGGAAAC | 57.1 |  |
| P2X_1_ | Sense | CTGTGAAGACGTGTGAGATCTTTGG | 60.7 | 463 |
|  | Antisense | TTGAAGAGGTGACGGTAGTTGGTC | 59.1 |  |
| P2X_2_ | Sense | GCTGCTCATCCTGCTCTACTTCGTGTGG | 68.0 | 399 |
|  | Antisense | GGGGTAGTGGATGCTGTTCTTGATGAGG | 64.0 |  |
| P2X_3_ | Sense | ATCAACCGAGTAGTTCAGC | 53.9 | 695 |
|  | Antisense | GATGCACTGGTCCCAGG | 53.2 |  |
| P2X_4_ | Sense | GGCCTTCCTGTTCGAGTACG | 58.3 | 596 |
|  | Antisense | TGGGAAGGATATTCCTCTTGC | 55.3 |  |
| P2X_5_ | Sense | CATGGGGCAGGCGGG | 55.8 | 598 |
|  | Antisense | GGGGAAACGGATGTGGTTCT | 55.4 |  |
| P2X_6_ | Sense | AGTTCAACTTCTCTAAGTCCAATGC | 58.1 | 470 |
|  | Antisense | CTCTATCCACATACAGCAGTAGC | 58.1 |  |
| P2X_7_ | Sense | CCCCGGCCACAACTACACCACGAGAAAC | 65.8 | 440 |
|  | Antisense | CCGAAGTAGGAGAGGGTTGAGCCGATG | 66.3 |  |
| P2Y_1_ | Sense | CCTGCGAAGTTATTTCATCTA | 53.4 | 318 |
|  | Antisense | GTTGAGACTTGCTAGACCTCT | 55.8 |  |
| P2Y_2_ | Sense | GCAGCATCCTCTTCCTCACCT | 60.0 | 502 |
|  | Antisense | CATGTTGATGGCGTTGAGGGT | 57.9 |  |
| P2Y_4_ | Sense | GGCATTGTCAGACACCTTGTA | 55.1 | 551 |
|  | Antisense | AAGGCACGAAGCAGACAGCAA | 61.4 |  |

(Continued on the next page)

Table S4 (continued)

| Targets | | Primer sequences | Tm*  (^o^C) | Size  (bp) |
| --- | --- | --- | --- | --- |
| P2Y_6_ | Sense | CGCTTCCTCTTCTATGCCAA | 57.5 | 480 |
|  | Antisense | GTAGGCTGTCTTGGTGATGTG | 56.2 |  |
| P2Y_11_ | Sense | ACTTCCTGTGGCCCATACTG | 56.3 | 499 |
|  | Antisense | GCTGTCCCCAGACACTTGAT | 56.3 |  |
| P2Y_12_ | Sense | CTAAGATTCTCTCTGTTGTCATCTG | 57.8 | 432 |
|  | Antisense | ACAGAGTGCTCTCTTTCACATAG | 57.9 |  |
| P2Y_13_ | Sense | TGTGTCGTTTTTCTTCGGTG | 54.4 | 578 |
|  | Antisense | TGCTGCCAAAAAGAGAGTTG | 55.8 |  |
| P2Y_14_ | Sense | CGCAACATATTCAGCATCGTGT | 58.9 | 102 |
|  | Antisense | GCTGTAATGAGCTTCGGTCTGAC | 60.2 |  |
| STIM1 | Sense | AGGCAGTCCGTAACATCCAC | 56.3 | 547 |
|  | Antisense | TGAAGAAGATGATGAAGGAC | 51.0 |  |
| Orai-1 | Sense | AGAGTTACTCCGAGGTGATG | 54.8 | 308 |
|  | Antisense | GCACAATCTCAACTCGGTC | 53.9 |  |
| TRPC1 | Sense | ATTTTGGAAAATTTCTTGGGATGT | 53.3 | 362 |
|  | Antisense | TTCATGATTTGCTATCA | 43.5 |  |
| TRPC3 | Sense | CAAGAATGACTATCGGAAGC | 53.5 | 201 |
|  | Antisense | GCCACAAACTTTTTGACTTC | 50.9 |  |
| TRPC4 | Sense | GGACTTCAGGACTACATCCA | 53.3 | 201 |
|  | Antisense | ACGCAGAGAACTGAAGATGT | 55.6 |  |
| TRPC5 | Sense | CCACCAGCTATCAGATAAGG | 54.2 | 159 |
|  | Antisense | CGAAACAAGCCACTTATACC | 52.6 |  |
| TRPC6 | Sense | TGAAGTGAAATCAGTGGTCA | 52.0 | 175 |
|  | Antisense | AAATTTCCACTCCACATCAG | 51.2 |  |
| TRPC7 | Sense | CATAGCCTATTGGATTGCTC | 53.2 | 176 |
|  | Antisense | GGTAGTCTGTGAATTTCG | 48.4 |  |
| CD39 | Sense | ACCATTCCCACGTCTTCACATTTG | 58.5 | 157 |
|  | Antisense | GTGGGCGGTGAACGAGAGAA | 59.1 |  |

(Continued on the next page)

Table S4 (continued)

| Targets | | Primer sequences | Tm*  (^o^C) | Size  (bp) |
| --- | --- | --- | --- | --- |
| CD73 | Sense | CACCAAGGTTCAGCAGATCCGC | 61.7 | 333 |
|  | Antisense | GTTCATCAATGGGCGACCGG | 58.3 |  |
| ALP** | Sense | GGACCATTCCCACGTCTTCAC | 56.6 | 137 |
|  | Antisense | CCTTGTACCCACGCCCATTG | 57.6 |  |
| GAPDH | Sense | GCAGGGGGGAGCCAAAAGGG | 60.7 | 567 |
|  | Antisense | TGCCAGCCCCAGCGTCAAAG | 62.8 |  |

*Each Tm was based on the Nearest Neighbor methods.

**ALP: alkaline phosphatase
