# Supplementary material for: NCI-H295R, a Human Adrenal Cortex-Derived Cell Line, Expresses Purinergic Receptors Linked to Ca2+-Mobilization/Influx and Cortisol Secretion
Source: PLoS One. 2013 Aug 8;8(8):e71022. doi: 10.1371/journal.pone.0071022 (PMC3738630; doi:10.1371/journal.pone.0071022)
Supplement: Text S1 — Supporting Information. (DOC) [file pone.0071022.s010.doc]

**Supporting Information**

HRP-conjugated primary antibodies for human proteins:
    The following primary human antibodies were used: anti-P2Y_1_ receptor, anti-TRPC6 channel, and anti-GAPDH (Abcam, Cambridge, England); anti-A_2A_ receptor, P2X_5_ receptor, P2X_7_ receptor, anti-P2Y_2_ receptor, P2Y_12_ receptor, P2Y_13_ receptor, anti-CD39, and anti-CD73 (GeneTex, San Antonio, TX); anti-A2B receptor (Enzo Life Sciences, New York, NY); anti-P2Y_6_ receptor and anti-TRPC1 channel (Osenses, SA, Australia); anti-P2Y_14_ receptor, anti-STIM1, and anti-alkaline phosphatase (Novus, Littleton, CO); anti-TRPC3 channel (Sigma-Aldrich, Saint Louis, MO); anti-Orai1 (AnaSpec, Fremont, CA); anti-TRPC5 channel (StressMarq, Victoria, Canada). Control peptide for anti-STIM1 (Novus, Littleton, CO). Primary antibodies were used in 0.001-1 μg/mL depending on each antibody.

Secondly antibodies:
    The following secondary antibodies were employed: peroxidase-conjugated anti-rabbit IgG and peroxidase-conjugated anti-mouse IgG (Jackson ImmunoResearch, West Grove, PA); and DyLight 488-labeled antibody to mouse IgG (KPL, Gaithersburg, MD). Secondary antibodies were used in 0.00125-0.01 μg/mL depending on each case.

Detection of ecto-alkaline phosphatase in H295R
In order to investigate the expression of ecto-alkaline phosphatase (ecto-ALP) on the H295R cell surface, DAPI (0.0005 mg/mL), a nucleus staining material, was added to H295R in culture and incubated for 5 min at room temperature.  The cells were then washed twice with PBS and fresh media containing anti-APL antibody (0.01 mg/mL) was added and incubated for 4 hr.  As a control, we used our anti-TRPC5 antibody (0.01 mg/mL) because TRPC5 is a cell surface-expressed protein like an ecto-ALP.  Furthermore, our antibody for TRPC5 is a mouse IgG as was the anti-ALP we used.  After cultivation with the antibodies, the media in the wells was discarded, and the cells washed twice with PBS.  Fresh media was then added to the wells followed by exposure to the DyLight 488-labeled 2ndary antibody, an anti-mouse antibody (0.004 mg/mL), and incubated for 1 hr.  After the incubation, the media in the wells was discarded, and the cells were washed twice with PBS before observation using fluorescent microscopy (Nikon, Tokyo, Japan) with the image acquisition/analysis system, AquaCosmos (Hamamatsu Photonics, Hamamatsu, Japan).

**Supporting Figure Legends**

**Figure S1. Correlation analysis between the fluorometric analysis and HPLC-RIA for cortisol quantifications.**

Amounts of cortisol in the culture medium quantified by the fluorometric analysis correlate with the results obtained by HPLC-RIA (r = 0.9340). In these tests, basal levels, those stimulated by 1000 μM 2MeS-ATP, by 500 μM db-cAMP, and by 100 μM forskolin were compared between the fluorometric analysis and HPLC-RIA (N = 4-6).

**Figure 2S.  Analysis of expression of ecto-alkaline phosphatase.**
Images of ALP (**1**) and TRPC5 (**2**) on the H295R cell surface. **1A** and **2A**: transparent images, **1B** and **2B**: images of Alexa 488 as protein expression, 1C and 2C: images of DAPI for nucleoli, and **1D** and **2D**: overlay of A, B, and C. For DyLight 488-labeled 2ndary antibody (Alexa Fluor 488) examination, cells were excited at 488 nm and the emission was observed through a 520 nm band path filter, respectively.  For DAPI, the cells were excited at 358 nm and the emission was observed through a 460 nm band path filter. TRPC5 (**2B**) expression is positive but ALP is not (**1B**) as ecto-ALP. All images were originally observed as monochromes and changed to pseudo-colors.  Scale bar indicate 50 μm.

**Figure S3. Time-dependent cortisol secretion assay in H295R.**

**A) Time-course of cortisol secretion in H295R.**

Time course study of cortisol secretion by 1 mM 2MeS-ATP in H295R. Data represent the Mean + SE (N = 4). The curve fitting in A was performed by the GraphPad Prism (GraphPad Software, La Jolla, CA).

**B) Cortisol secretion following short-term incubation in H295R**

Short-term incubation procedures were performed using 2MeS-ATP.  The media was removed from the well at 1, 2, 4, 8, 12, 24, or 36 hr after application of the agents. Once the media was removed, the wells were washed and refilled with the same volume (1 mL) of fresh media and maintained in incubation without any agents until 48 hr. Graph shows cortisol secretion at 48 hr after the application. Data represent the Mean + SE (N = 4).

**Figure S4. Effects of purinergic agonists on GC secretion under Ca^2+^-free conditions in H295R.**No agonists tested under Ca^2+^-free conditions (2 mM EGTA) induced significant GC secretion. For comparison, GC secretion by 2MeS-ATP under standard condition (1.2 mM Ca^2+^) is shown as Mean + SE (N = 4).

**Figure S5. Comparative assay for the effect of an L-type VDCC blocker on 2MeS-ATP- or AngII-induced glucocorticoid secretion.**

Effects of nifedipine, an L-type VDCC-blocker, on 2MeS-ATP or AngII-induced glucocorticoid secretion in H295R.  The cells were incubated at 37°C for 48h.  Each histogram represents the Mean + SE (N = 4).  ‘*’, ‘**’, statistical significance at *p* < 0.05, *p* < 0.01, respectively.
